# Recovery of depth perception in adults with abnormal binocular vision^☆^

**DOI:** 10.1016/j.visres.2026.108783

**Published:** 2026-02-13

**Authors:** Jian Ding, Michelle Y. Ma, Hilary H. Lu, Benjamin T. Backus, Dennis M. Levi

**Affiliations:** a Herbert Wertheim School of Optometry and Vision Science and the Helen Wills Neuroscience Institute University of California, Berkeley, CA 94720-2020, USA; b Vivid Vision, Inc., San Francisco, CA, USA; c State University of New York College of Optometry, NY, USA

**Keywords:** Perceptual learning, Stereo training, Virtual Reality, Amblyopia, Anisometropia, Strabismus

## Abstract

Our goal in this study was to determine the efficacy, time course and mechanisms underlying the improvement of stereo vision using 3D virtual reality (VR) games to improve depth perception in adults with a history of abnormal early visual experience due to strabismus, anisometropia and/or amblyopia. Participants engaged in 30 home-based training sessions, supplemented by 5 in-lab assessments to monitor progress. The training protocol utilized a diverse set of VR games targeting stereoacuity, anti-suppression, and binocular alignment. The stereo training games employed various depth cues to facilitate stereo vision recovery. Clinical, psychophysical and virtual reality tests were conducted to evaluate changes in stereoacuity, binocular balance, and interocular alignment. Results revealed significant improvements in stereoacuity for most participants following training, whereas outcomes for binocular balance and ocular alignment were more variable. Together, these findings suggest that VR-based training can reliably enhance stereoacuity in adults with abnormal visual histories, though individual variability highlights the need for personalized approaches and further investigation of underlying mechanisms.

## 1. Introduction

Under everyday viewing conditions, with both eyes open, a key problem for individuals with a history of abnormal early visual experience due to strabismus (mis-aligned eyes) or anisometropia (unequal refractive error in the two eyes) is impaired stereopsis (Webber 2018). Stereopsis is the percept of depth that arises from the difference between the right and left retinal images. Individuals with reduced stereopsis may have difficulty with sports, reaching for and grasping objects (Grant et al., 2007; Melmoth et al., 2009; Niechwiej-Szwedo, Chandrakumar, et al., 2012; Niechwiej-Szwedo, Kennedy, et al., 2012; Suttle et al., 2011; Vedamurthy et al., 2016) and locomoting in complex terrains (Bonnen et al. 2021).

Amblyopia is a common visual impairment stemming from abnormal visual development in early childhood, generally associated with strabismus, anisometropia or both, and it results in decreased visual acuity in one eye, and reduced binocular vision and depth perception. Traditional treatments such as patching or atropine penalization have primarily focused on improving visual acuity in the amblyopic eye. However, stereo training, which aims to enhance the collaboration and interaction between both eyes, has emerged as a promising approach for addressing the binocular deficits associated with amblyopia and its associated conditions (Levi, 2022). By encouraging the brain to utilize both eyes in tandem, stereo training seeks to improve stereopsis (depth perception) and facilitate a more holistic visual rehabilitation.

Challenging the traditional view of amblyopia as solely a monocular disorder, recent research emphasizes the role of binocular dysfunction in its development (Meier and Giaschi 2017, Birch et al. 2021, Brin et al. 2021, Banko et al. 2022) (see also Hess (2025) this issue). This shift in understanding has paved the way for binocular treatment approaches like stereo training, which encompasses various methods with unique advantages. Perceptual learning, involving repeated practice of depth detection visual tasks, can significantly enhance stereopsis in adults with abnormal binocular vision, even in those previously deemed stereoblind (Astle and McGraw, 2011; Chopin et al., 2021; Ding & Levi, 2011; Xi et al., 2014). Dichoptic training utilizes techniques, presenting the background image to both eyes and specific targets only to the weaker eye, adjusting contrast or luminance between the eyes, and employing 3D technology (Vedamurthy et al. 2015, Banko et al. 2022). This approach can improve not only stereopsis but also visual acuity and contrast sensitivity (Li et al. 2013, Vedamurthy et al. 2015). Finally, vergence and accommodation training enhances the ability of the eyes to converge and accommodate on objects at varying distances, addressing potential difficulties with reading, near work, and depth perception (Thiagarajan and Ciuffreda 2013, Thiagarajan and Ciuffreda 2014a, Thiagarajan and Ciuffreda 2014b, Ciuffreda and Thiagarajan 2022).

Despite these positive outcomes, the widespread implementation of stereo training faces challenges. Treatment results can be inconsistent, influenced by factors such as the severity of amblyopia, the patient's age, and their level of adherence to the training regimen. Furthermore, traditional stereo training methods often suffer from limited accessibility, typically requiring patients to undergo training in a specialized facility under direct supervision. However, the rapid advancements in virtual reality (VR) technology have opened new possibilities for vision therapy. VR's immersive and interactive nature provides a unique environment for enhancing traditional ophthalmology and optometry practices (Žiak et al. 2017, Levi 2023, Leal-Vega et al. 2024). VR has been successfully used to treat binocular vision disorders such as amblyopia and strabismus (Žiak et al. 2017, Backus et al. 2018, Coco-Martin et al. 2020, Levi 2023). Its immersive nature makes it more engaging than traditional therapy methods, especially for children, leading to improved compliance with treatment regimens (Eastgate et al. 2006, Coco-Martin et al. 2020, Godinez et al. 2021, Levi 2023). Backus et al. (2018), Vivid Vision, among others (Levi 2023, Leal-Vega et al. 2024), have developed VR games that allows clinicians to meticulously control the visual environment, including disparity, contrast, luminance, and content presented to each eye independently. These games can simulate real-world environments, allowing patients to practice visual skills in a safe and controlled setting and promoting the transfer of training benefits to everyday activities (Levi 2023, Leal-Vega et al. 2024). VR systems can also provide objective data on patient performance, enabling clinicians to track progress and tailor treatment plans (Backus et al. 2018, Leal-Vega et al. 2024).

While VR vision therapy holds immense potential, challenges persist. These include vergence-accommodation conflict, where the fixed focal distance of VR headsets can create visual discomfort and reduce performance, and cyber sickness, with some individuals experiencing motion sickness or discomfort while using VR (Akizuki et al. 2005, Banks et al. 2016, Harris et al. 2019, Coco-Martin et al. 2020, Tychsen and Thio 2020, Levi 2022). Addressing these challenges requires standardized protocols and validation studies to establish the efficacy and reliability of VR-based vision therapy (Coco-Martin et al. 2020). Despite these challenges, VR vision therapy is a rapidly evolving field with immense potential to revolutionize the treatment of binocular vision disorders and other visual impairments (Coco-Martin et al. 2020, Levi 2023). Continued advancements in VR technology, coupled with ongoing research, are expected to address current challenges and lead to the development of even more effective and accessible treatment options.

Our goal in this study was to determine the efficacy, time course and mechanisms underlying the improvement of stereo vision. To accomplish this we employed a bespoke stereo training program (developed in collaboration with Vivid Vision, Inc.) that leverages the power of 3D VR games to improve stereopsis in individuals with strabismus, anisometropia and amblyopia. This innovative approach combines personalized home-based training with in-lab assessments to target multiple aspects of depth perception, including stereoacuity, anti-suppression, and eye alignment. Preliminary findings have revealed fascinating patterns of improvement and decline in depth perception over time, highlighting the dynamic nature of visual rehabilitation. Further exploration will be crucial in refining VR-based stereo training protocols and maximizing its potential for comprehensive amblyopia treatment.

## 2. Methods

**Fig. 1.**
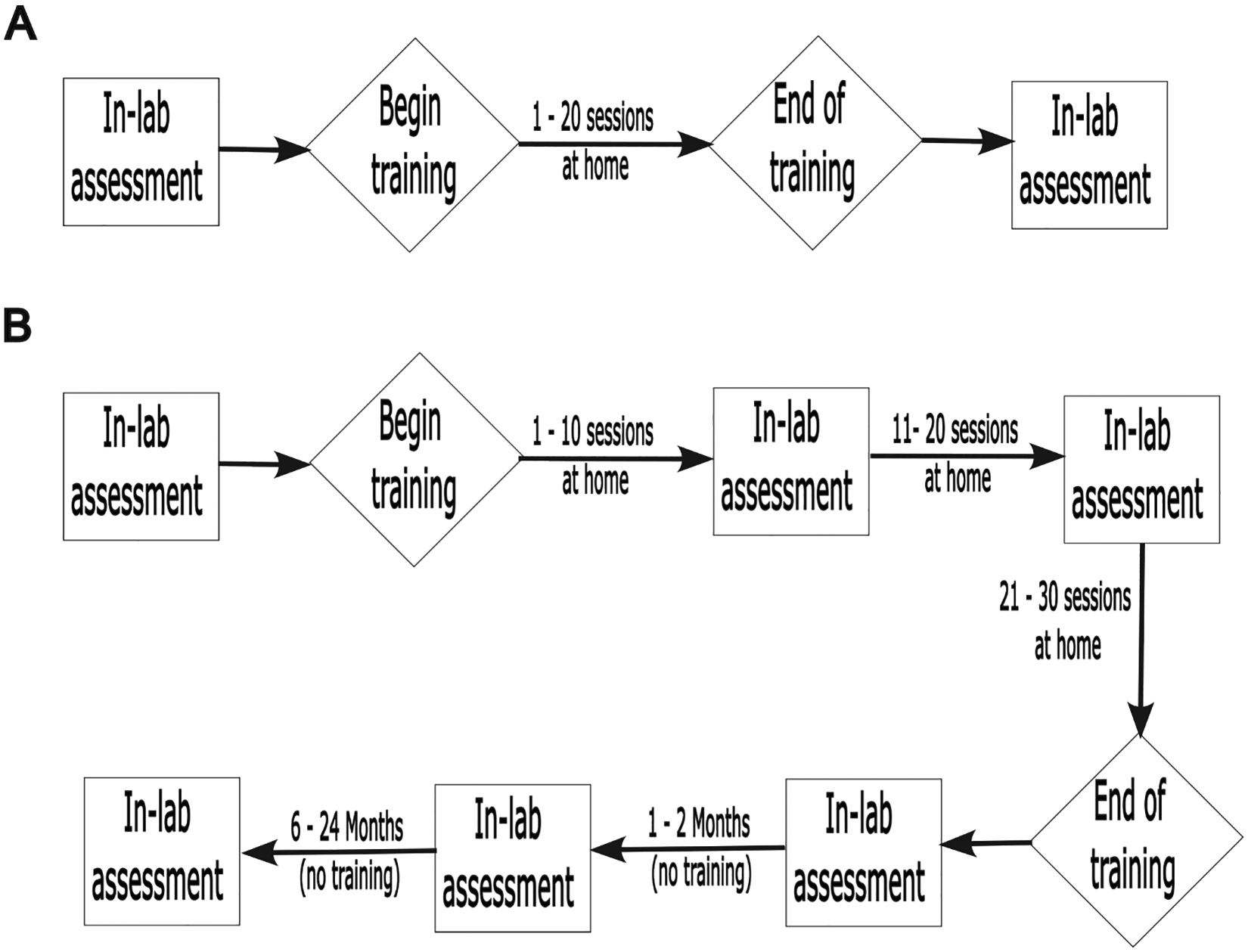
Schematic timeline of training and testing sessions. A. Training and testing protocol for control participants (C2 and C3). These participants completed 20 home-based sessions and two in-lab assessments (pre- and post-training) to provide an internal reference for expected ceiling performance, task tolerability, and verification of testing procedures. B. Training and testing protocol for all other participants. Participants completed baseline in-lab assessments (clinical stereo tests, psychophysical measures, and VR tests) prior to training. Training consisted of 30 one-hour home-based VR sessions, with in-lab assessments after 10, 20, and 30 sessions to monitor progress. A subset of participants (A2, A3, A5, A6) also completed follow-up assessments 1– 24 months post-training to evaluate longer-term outcomes.

Vivid Vision offers a diverse range of child-friendly VR games and activities designed to address various aspects of binocular vision dysfunction. These games leverage the immersive nature of virtual reality to provide engaging and effective vision therapy experiences. In collaboration with Vivid Vision, we have developed a novel, personalized stereo training program using 3D VR games to improve stereopsis in individuals with abnormal binocular vision. As an initial step, we tested a *preliminary training protocol* (Fig. 1A) with two normal control participants (C2 and C3). They completed 20 home-based sessions and two in-lab assessments (pre- and post-training) to establish an internal reference for ceiling performance, task tolerability, and verification of testing procedures. We then refined this into the *final training protocol* (Fig. 1B), consisting of 30 personalized home-based sessions, each lasting approximately 1 h (including a short break or time for switching between activities) and 4–––7 in-lab assessments (approximately, each lasting about 2 h each). Participants were instructed to complete at least three home-based sessions per week, with a maximum of one session per day. Each session includes seven VR games targeting stereoacuity, anti-suppression, and vergence facility, along with three VR tests assessing stereoacuity, binocular balance, and interocular alignment. In-lab assessments include clinical measures of visual acuity and stereoacuity, as well as psychophysical measurements of equivalent internal disparity noise and detection efficiency for two kinds of binocular disparity stimulus – a single-plane Random Gabor Patch (RGP) stereogram and a split-plain RGP stereogram. Assessments were conducted once before training, twice during training (after 10 and 20 sessions), and one after training. Follow-up assessments post-training are conducted when possible. A third normal control participant (C1) completed the final training protocol to provide a full evaluation.

### 2.1. Virtual reality (VR) games for binocular vision therapy

The following shows the playing order of a home-based training session:

1. Prism Tuner Prism Tuning Test 2.0 min
2. Stereoacuity Test 2.0 min
3. Interocular Balance Test 2.0 min
4. Bullseye Stereoacuity Game 2.5 min
5. Bubbles Stereoacuity Game 2.5 min
6. Breaker Anti-suppression Game 2.5 min
7. Pepper Picker Anti-suppression Game 5.0 min
8. Barnyard Bounce (BO) Vergence & Anti-suppression Game 2.5 min
9. Step Vergence (BI) Vergence Game 2.5 min
10. Barnyard Bounce (BI) Vergence & Anti-suppression Game 2.5 min
11. Step Vergence (BO) Vergence Game 2.5 min
12. Bullseye Stereoacuity Game 2.5 min
13. Bubbles Stereoacuity Game 2.5 min
14. Jump Duction (BIBO) Vergence Game 2.5 min
15. Bubbles Stereoacuity Game 2.5 min
16. Bullseye Stereoacuity Game 2.5 min
17. Stereoacuity Test 2.0 min

**Fig. 2.**
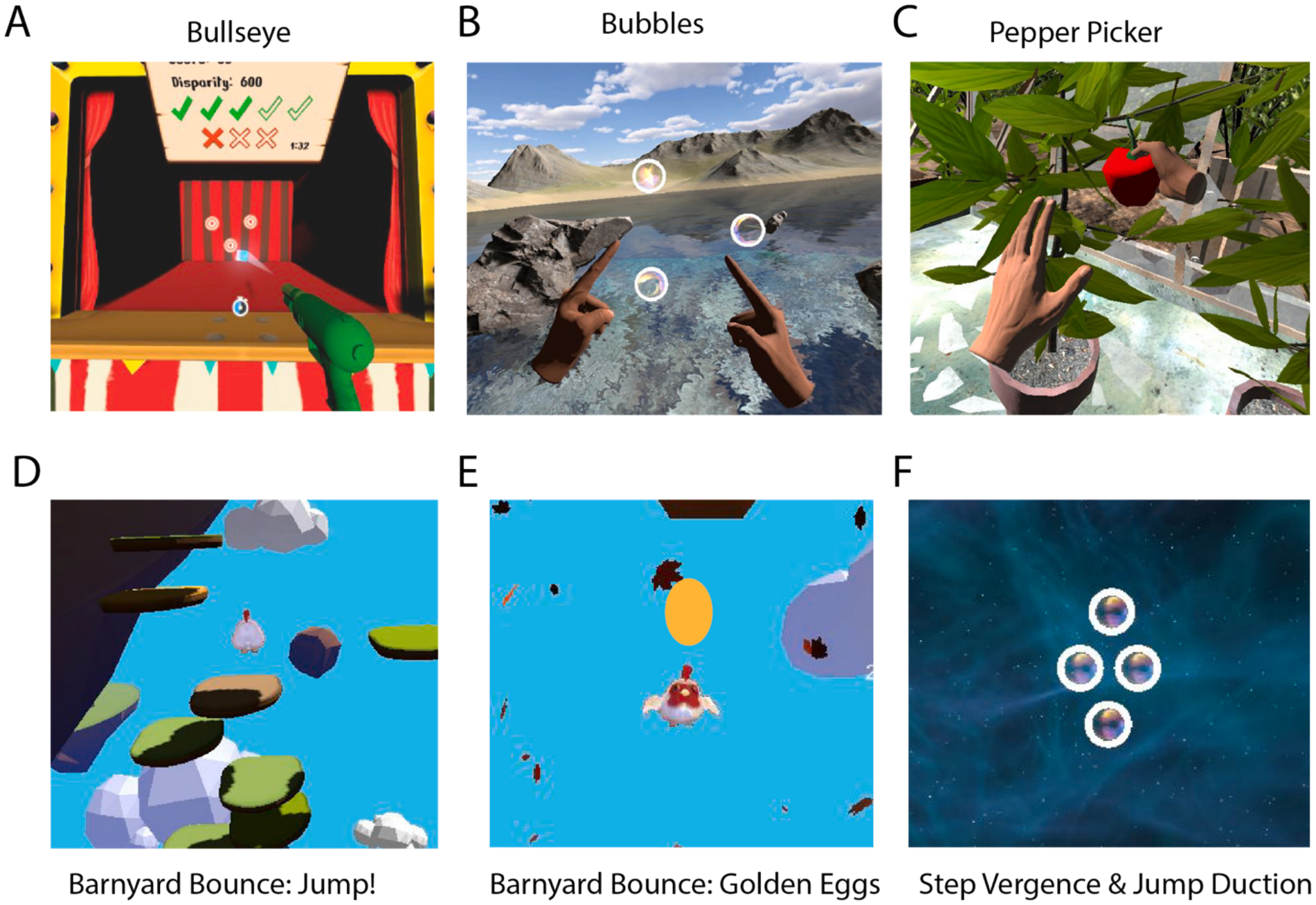
Scenes from Vivid Vision home-based binocular vision therapy games. **(A) Bullseye:** The player uses a virtual water gun to shoot targets in a carnival booth. The correct target is the closest one. This game helps improve eye coordination, aiming, and depth perception. **(B) Bubbles:** The player uses virtual fingers to pop bubbles in order from closest to farthest, enhancing depth perception and eye tracking skills. **(C) Pepper Picker:** The player searches for a pepper in a virtual greenhouse and uses a virtual hand to reach for it and place it in a basket. This game challenges depth perception and hand-eye coordination in a 3D environment. The visual targets and the virtual hands are presented to different eyes, requiring the player to fuse the images from both eyes to successfully complete the task. **(D) Barnyard Bounce:** The player uses a controller to vary the chicken's position while it jumps up to land on a floating rock. As the chicken jumps, the vergence demand changes to either a preselected Base-In (BI) or Base-Out (BO) direction, training the eyes to converge or diverge accurately. **(E) Barnyard Bounce (Golden Egg):** Occasionally, the chicken flies to collect a dichoptically visible golden egg. The player must align the chicken with the egg as it flies, testing vergence accuracy. **(F) Step Vergence & Jump Duction:** The targets are stereoscopic, with one appearing to pop out of the scene. In Step Vergence, the vergence demand changes to either a preselected Base-In (BI) or Base-Out (BO) direction in steps. In Jump Duction, the vergence demand changes randomly in both directions, training the eyes to quickly and accurately adjust to varying vergence demands.

Two games specifically target stereoacuity. Bullseye (Fig. 2A) is a perceptual learning task designed to enhance distance stereo vision. Players are presented with multiple targets at varying depths and challenged to identify and shoot the nearest one. The target disparity adjusts dynamically based on performance, decreasing after a correct response and increasing after an incorrect response. Bubbles (Fig. 2B) focuses on near stereo training and is also designed to improve eye-hand coordination. In this game, players pop bubbles floating at different depths in order from closest to farthest. The difficulty level, again, adjusts based on performance. Each stereo game was played in three 2.5-minute blocks, totaling 15 min dedicated to direct stereo training.

Our bespoke version of Bubbles is a cue scaffolding game (Vedamurthy et al. 2016, Godinez et al. 2021, Levi 2022) in which the player uses stereo and motion parallax to pick the closest of N = 2 or more bubbles. At a given level of disparity, the reliability of motion parallax is reduced until only binocular disparity remains. To reduce the reliability of the motion parallax cue, the amplitude of lateral head motion is restricted. When the player moves his/her head outside of the allowed area, the scene goes dark and he/she sees an arrow directing the subject to move their head back into the allowed volume. Amplitude of head motion is reduced gradually to zero during the session, independent of binocular disparity, which is the staircase variable. Breaker also utilized cue scaffolding, with object looming and motion parallax supplying additional depth cues. To reduce the reliability of the looming cue, the amplitude of retinal size change for the ball within the game was reduced over time, which incidentally caused the ball to change in apparent size by shrinking as it approached and expanding as it receded, as expected due to size constancy.

Two games specifically target anti-suppression. Breaker (not shown) enhances tracking abilities and improves eye-hand coordination. Players use a paddle (visible to the dominant eye) to hit a ball (visible to the non-dominant eye) and break bricks while avoiding penalty markers. Difficulty adjusts automatically over time by varying ball speed, size, and brick complexity based on player performance. Pepper Picker (Fig. 2C) also focuses on anti-suppression and adds a figure-ground component. In this game, the dominant eye sees the virtual hands while the non-dominant eye sees the peppers. Players search for specific peppers in a greenhouse and place them in a basket, which requires integrating information from both eyes. Difficulty adapts through changes in pepper size, color, and plant layout.

Three vergence training games aim to improve the ability of both eyes to focus on objects at varying distances. In Barnyard Bounce (Fig. 2D), players guide a chicken onto ascending platforms by varying its position. Vergence demands increase in a preselected direction (Base-In or Base-Out) during jumping. If the vergence demand exceeds the player's capabilities, diplopia (double vision) occurs, providing immediate feedback on their vergence limits. Barnyard Bounce also includes fixation disparity checks. In these checks, the chicken is visible to the dominant eye while a golden egg appears to the non-dominant eye against a background of binocular leaves (Fig. 2E). Unlike the anti-suppression games *Breaker* and *Pepper Picker*, where alignment is only implicit, *Barnyard Bounce* explicitly requires the player to align the chicken with the golden egg. This task promotes proper fixation and helps reveal suppression or other fixation difficulties. In Step Vergence & Jump Duction (Fig. 2F), four targets are presented, and the player must select the one that appears closer. Step Vergence trains smooth vergence adjustments by having players follow targets that change vergence in steps in either the Base-In or Base-Out direction. Jump Duction challenges quick vergence adjustments with targets jumping between the two directions. The vergence demand adjusts dynamically based on performance, increasing with a correct response and decreasing with an incorrect response. Barnyard Bounce and Step Vergence each consist of two blocks: one with a virtual prism base-out (BO) and the other base-in (BI). Jump Duction consists of one block, with a virtual prism randomly selected as base-in or base-out for each trial. These Vivid Vision games simulate prismatic demand by shifting virtual images, offering controlled vergence training without physical prisms, which enhances binocular vision and comfort.

Each of these VR games emphasizes a particular set of visual skills. Bubbles isolates stereoscopic depth perception using a stereoacuity task, while the other games integrate multiple skills that the player works on concurrently. Different skills receive different emphasis within different games. These skills include: (1) Binocular fusion: The ability to combine the images from the two eyes into a single, unified percept. This is essential for all aspects of binocular vision. (2) Stereoscopic depth perception: The ability to perceive depth based on the slight differences between the images seen by the two eyes. (3) Hand-eye coordination: The ability to coordinate visual information with hand movements. This is important for tasks such as reaching and grasping. (4) Peripheral awareness: The ability to attend to visual information in the periphery of the visual field. In *Pepper Picker*, the primary task is to select the correct colored peppers from the garden. However, because target peppers often appear across a wide area of the screen rather than just at the center, players must scan beyond central vision to find them efficiently. Distractor peppers are also scattered throughout, requiring players to quickly distinguish targets from non-targets—an action that draws on peripheral awareness to detect items appearing outside the point of fixation. (5) Vergence eye movement control: The ability to accurately converge or diverge the eyes to focus on objects at different distances.

Three VR-based visual tests (not shown) were also conducted during each training session. The Stereoacuity Test (Denkinger et al. 2023) is performed before and after each session to track changes in stereoacuity. The player is presented with four circular targets on a moving surface, one of which appears to float off the surface. The task is to identify which target is floating. With each correct response, the targets become smaller and the disparity decreases. With each incorrect response, the targets become larger and the disparity increases. The Interocular Balance Test (not shown) is performed before a session to assess the balance of visual input between the two eyes, aiming to identify suppression issues that may hinder binocular vision. This test uses dichoptic presentation, showing each eye a slightly different image. To detect imbalances, the luminance (brightness) or contrast of the dominant eye’s image is gradually reduced. The level of reduction needed to achieve balance quantifies the degree of suppression or dominance. Repeated testing allows monitoring of interocular balance over time, helping to gauge training effectiveness. The Prism Tuner Test (Not shown) is also conducted before a session to optimize the virtual prism settings. It tests only at a distance of 200 cm, ensuring that accommodative and vergence demands are properly matched in the headset. Similar to a Maddox rod test, the player aligns a vertical or horizontal line with a spot. The cyclovergence estimate uses a pair of horizontal lines similar to a double-Maddox test. The test’s goal is to determine the minimal amount of virtual prism correction needed during the treatment session.

### 2.2. Psychophysical visual tests

#### 2.2.1. Binocular disparity thresholds

**Fig. 3.**
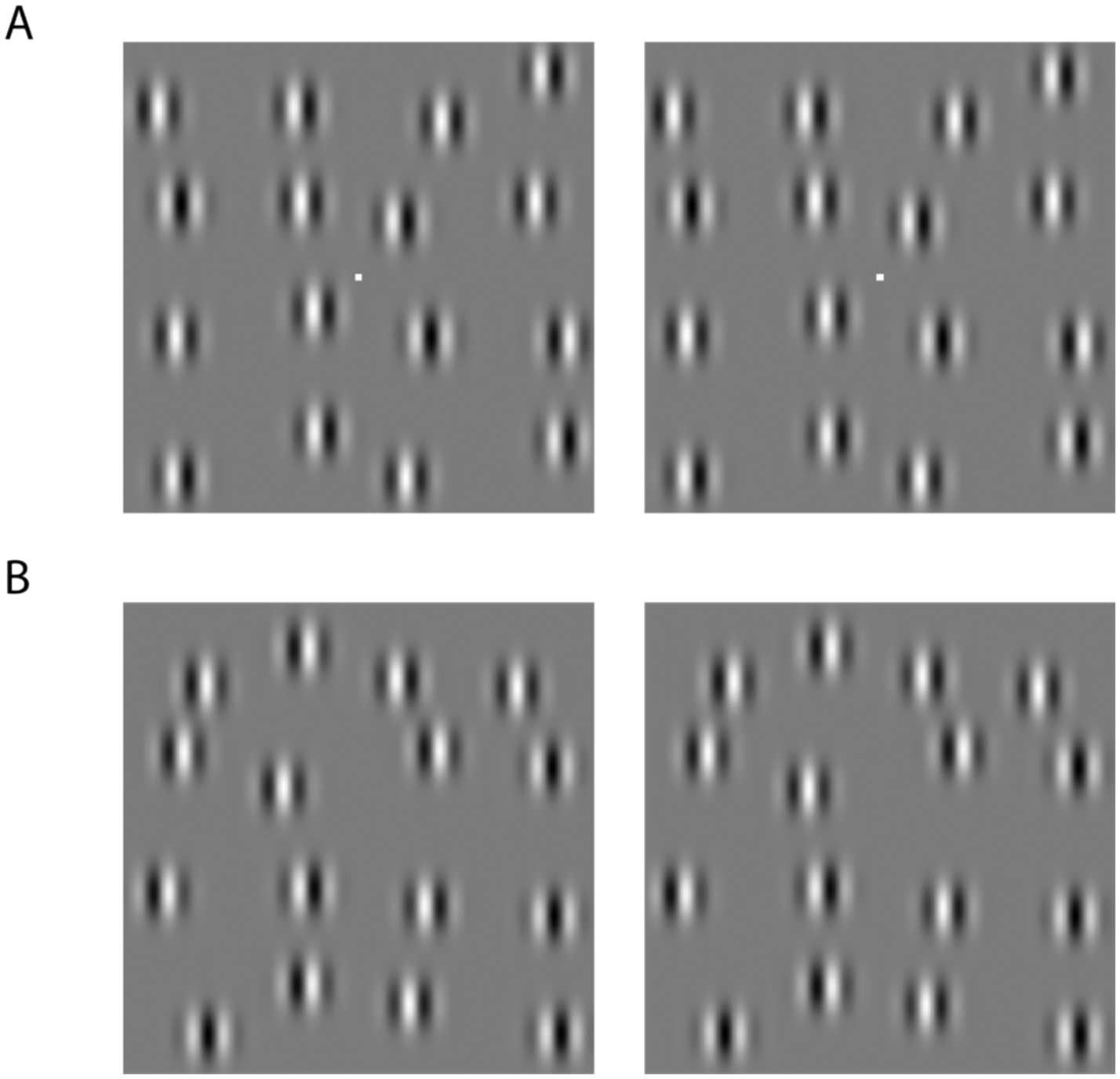
Random-Gabor-Patch (RGP) stereograms used in the single-plane and split-plane depth tasks. Each stereogram consisted of 16 vertical Gabor patches with random positions and phases but a fixed spatial frequency (0.75 cpd). The left- and right-eye images were identical except for corresponding patch pairs, which were shifted in opposite directions according to Gaussian horizontal and vertical distributions, producing random binocular disparities. Mean vertical disparity was always zero; mean horizontal disparity was non-zero. A. Single-plane condition. All patches had the same mean horizontal disparity (crossed or uncrossed) across the entire display. A fixation point (a bright spot of 8 × 8 arcmins) was included. B. Split-plane condition. The upper and lower halves of the array carried crossed and uncrossed mean horizontal disparities, respectively. No fixation point was included. In this example, with uncrossed fusion, the bottom region appears nearer in depth.

Random-Gabor-Patch (RGP) stereograms (Fig. 3) were used as stimuli in two psychophysical depth-discrimination tasks: a single-plane task and a split-plane task. Each stereogram contained 16 vertically oriented Gabor patches with random spatial positions and phases, a fixed carrier spatial frequency of 0.75 cpd, and a patch radius of 2.0°, all arranged within a 14.1° × 14.1° square. Patch contrast was set to 100% in both eyes. Additional methodological details are provided in Ding, Lu et al. (2024)).

RGP stereograms convey depth signals through a narrow spatial-frequency-and-orientation channel and contain no monocular cues or fusion-facilitating features. As such, binocular depth perception requires solving a relatively complex correspondence problem (Chopin et al., 2021). The two eyes viewed identical patch arrays except for corresponding pairs that were subject to equal-and-opposite random shifts in the two eyes. These shifts were drawn from two Gaussian distributions (horizontal and vertical), introducing external disparity noise. The mean vertical disparity was always zero, whereas the mean horizontal disparity was non-zero and served as the depth signal.

In the single-plane task (Fig. 3A), all patches shared a mean horizontal disparity that was either crossed or uncrossed across the entire display. A fixation point was added to reduce the influence of the absolute-disparity anomaly (Chopin et al. 2016; Ding, Lu & Levi, 2024). Thresholds were measured with a two-interval forced-choice (2IFC) paradigm: each trial consisted of two intervals (1 s each, separated by 0.5 s), one crossed and the other uncrossed, and observers indicated which interval appeared closer.

Including a fixation point in the single-plane stimulus created a reference in depth, and presumably observers performed the single-plane task by comparing each plane to the fixation point. However, our previous study (Ding et al. 2024) found that depth perception of an entire stereogram relative to the fixation plane operates via a different mechanism than the relative disparity between two parts of the stereogram, regardless of whether a fixation point is present or not. Although performance in single-plane disparity detection is much poorer without a fixation point, the internal disparity noise remains similar whether or not a fixation point is included. The difference in performance can be attributed to varying detection efficiency (details see Ding et al. (2024)). As discussed in Ding et al. (2024), single-plane disparity detection, even without a visible reference mark, may rely on memory-based references, with the ability improving through practice (Chopin et al. 2016). Introducing a visible reference mark can reduce the absolute disparity anomaly (Chopin et al. 2016, Ding et al. 2024) and improve performance. The fixation point was small and did not have much contrast energy in the carrier spatial frequency of the Gabor, so the visual system may have used a different mechanism to measure the relative disparity between the fixation mark and the single-plane stimuli than between the two halves of the split-plane stimulus. The fixation mark has minimal impact on reducing internal disparity noise compared to absolute disparity discrimination (Ding et al. 2024), so one possibility is that the absolute disparities of the fixation mark and single-plane array were compared relatively late during visual processing, as compared to the split-plane stimulus.

In the split-plane task (Fig. 3B), the upper and lower halves of the array carried opposite signs of mean horizontal disparity (crossed vs. uncrossed). Because performance relied on relative disparity, no fixation point was included. Thresholds were measured with a two-alternative forced-choice (2AFC) judgment in a single 1-s interval, with observers reporting which half of the stereogram appeared closer. In both tasks, the stimulus remained static during each presentation.

Stimuli were displayed on a 22-inch NEC MultiSync CRT monitor (1920 × 1440 resolution, 75-Hz refresh rate). The setup used a System76 Mini running Linux and Matlab (MathWorks, Inc.) with Psychtoolbox (Brainard, 1997; Pelli, 1997). A custom electronic circuit (Li et al., 2003) provided 14-bit grayscale precision. Luminance gamma correction was performed and verified using a Minolta LS-110 photometer: minimum luminance was 0.2 cd/m^2^, maximum was 74.2 cd/m^2^. Displays were viewed through a custom four-mirror stereoscope at an optical viewing distance of 68 cm.

#### 2.2.2. Equivalent internal disparity noise and efficiency

To examine learning effects on equivalent internal disparity noise and efficiency, we measured single- and split-plane disparity thresholds with added external disparity noise before, during, and after VR stereo training. This is the same disparity discrimination task described above, but with external disparity noise added to the stimulus. Equivalent internal disparity noise reflects the level of internal noise within the visual system that limits disparity processing, capturing the variability or uncertainty in an observer’s binocular disparity signals. Efficiency quantifies how effectively the observer uses the available disparity information. By comparing thresholds across different levels of external disparity noise, we can determine whether limitations in stereoscopic performance arise primarily from (1) elevated internal noise (a noisier disparity representation) or (2) reduced efficiency (less effective use of the disparity signal) (Ding, Lu & Levi, 2024). This external-noise approach provides mechanistic insight into the sources of impairment that cannot be inferred from threshold measurements alone. However, this task was very challenging for trainees with abnormal binocular vision. Although it was administered at every in-lab visit, only a subset of trainees were able to produce meaningful data after regaining measurable stereovision at different time points. As a result, the detailed analyses are presented in Supplementary Information B.

#### 2.2.3. Psychometric functions

The method of constant stimuli (MOCS) was employed to measure disparity thresholds. Data for one spatial frequency channel (0.75 cpd) were collected in blocks of 70–100 trials, each containing 7–10 disparities with 10 repetitions per level. A brief preliminary run was conducted to familiarize observers with the task and to determine an appropriate disparity range. When lab time permitted, 100-trial blocks with 10 disparities were run to measure both the minimum (Dmin) and maximum (Dmax) disparity thresholds; when time was limited, 70-trial blocks with 7 disparities were used to estimate only Dmin. The Dmax thresholds were excluded from further analyses.

To model the relationship between response probability and binocular disparity, data were fitted using *maximum likelihood estimation* with a psychometric function defined as the sum of two cumulative Gaussian functions—one increasing with disparity and the other decreasing. The Dmin threshold was defined as the disparity corresponding to a 71% correct response rate in the increasing component. Due to limited sampling at large disparities, some Dmax thresholds could not be reliably estimated. Figs. 5–7 show the fitted psychometric functions for trainees A2, A3, and A6 across all in-lab assessments.

To ensure the reliability and adequacy of the fitted psychometric functions, we implemented a bootstrap-based quality control procedure. Each psychometric function, derived from 10 repetitions per MOCS level, was resampled 2,000 times using bootstrap analysis. For each resample, the psychometric function was refitted, and the distribution of threshold estimates was used to compute *95% confidence intervals (CIs)*. Fits were considered reliable if the bootstrap CI was narrow relative to the mean threshold estimate. In addition, all individual fits were visually inspected to confirm close correspondence between observed data points and fitted curves. As shown in Figs. 5–8, most psychometric fits met these reliability criteria.

### 2.3. Statistical analysis

Because several trainees exhibited unmeasurable disparity thresholds prior to training, traditional parametric analyses were not appropriate. To evaluate training-related improvements, we adopted a combination of non-parametric and categorical approaches. For participants with unmeasurable thresholds, the upper limit of the testing range (e.g., 400 arcsecs for clinical test) was assigned as a *ceiling value*, and changes in disparity thresholds across sessions were assessed using the *Wilcoxon signed-rank test*, which accommodates censored and non-normally distributed data. In addition, to examine whether training increased the number of participants with measurable stereopsis, we applied *McNemar’s test* to compare the proportion of trainees who transitioned from “no measurable stereopsis” to “measurable stereopsis.” All analyses were conducted in MATLAB (MathWorks).

#### 2.3.1. Trainees

Sixteen trainees with abnormal binocular vision signed an informed consent form and participated in the training program. Two trainees withdrew due to experiencing headaches when playing VR games, and three trainees withdrew for other reasons (e.g., too busy). Table 1 shows the visual conditions of the eleven trainees who completed the entire program, along with their stereoacuities before and after training, as measured by the clinical stereo circle test (Randot Stereotest, Stereo Optical Co., Inc.), which has a measurable range of 20–400 arcseconds. Three normal controls with normal or corrected-to-normal visual acuity also signed an informed consent form and participated in the program. They were pre-screened for stereoacuity of 20 arcseconds or better, using the same stereo test. The control group (n = 3) was included as a small normative benchmark rather than a fully powered comparison group. Their role was to provide an internal reference for expected ceiling performance, task tolerability, and verification of testing procedures. The experimental protocol was approved by the internal board of the ethics committee (IRB) of University of California, Berkeley, according to the guidelines and regulations for human subject research.

## 3. Results

Table 1 reports clinical stereoacuity for all 11 trainees and three normal controls before and after training. Only three trainees demonstrated quantifiable stereo thresholds prior to training, which constrained our ability to track change using this metric. For the majority of trainees, the key observation was the *emergence of measurable stereopsis after training*, rather than fine-grained threshold shifts.

Table 2 summarizes the ocular alignment measurements obtained before and after training for four trainees with strabismus, assessed using the Vivid Vision Prism Tuning Test (Prism Tuner). This test quantifies the amount of virtual prism required to achieve binocular alignment. Similar to dissociative clinical methods such as the Maddox rod, the test first presents dissociated stimuli to estimate vertical, cyclo-, and horizontal deviations. It then introduces varying amounts of virtual horizontal prism to determine the minimal prism necessary for alignment. Positive misalignment values indicate a need for Base-Out (horizontal) or Base-Down (vertical) prism correction, whereas negative values indicate a need for Base-In (horizontal) or Base-Up (vertical) correction. However, because the test was administered using a stand-alone VR headset without an external display, the experimenter could not visually monitor task performance. As a result, the VR-based alignment measurements showed substantial day-to-day variability (see Supplementary Information B), making some of the alignment estimates difficult to interpret.

### 3.1. Vivid vision stereoacuity

**Fig. 4.**
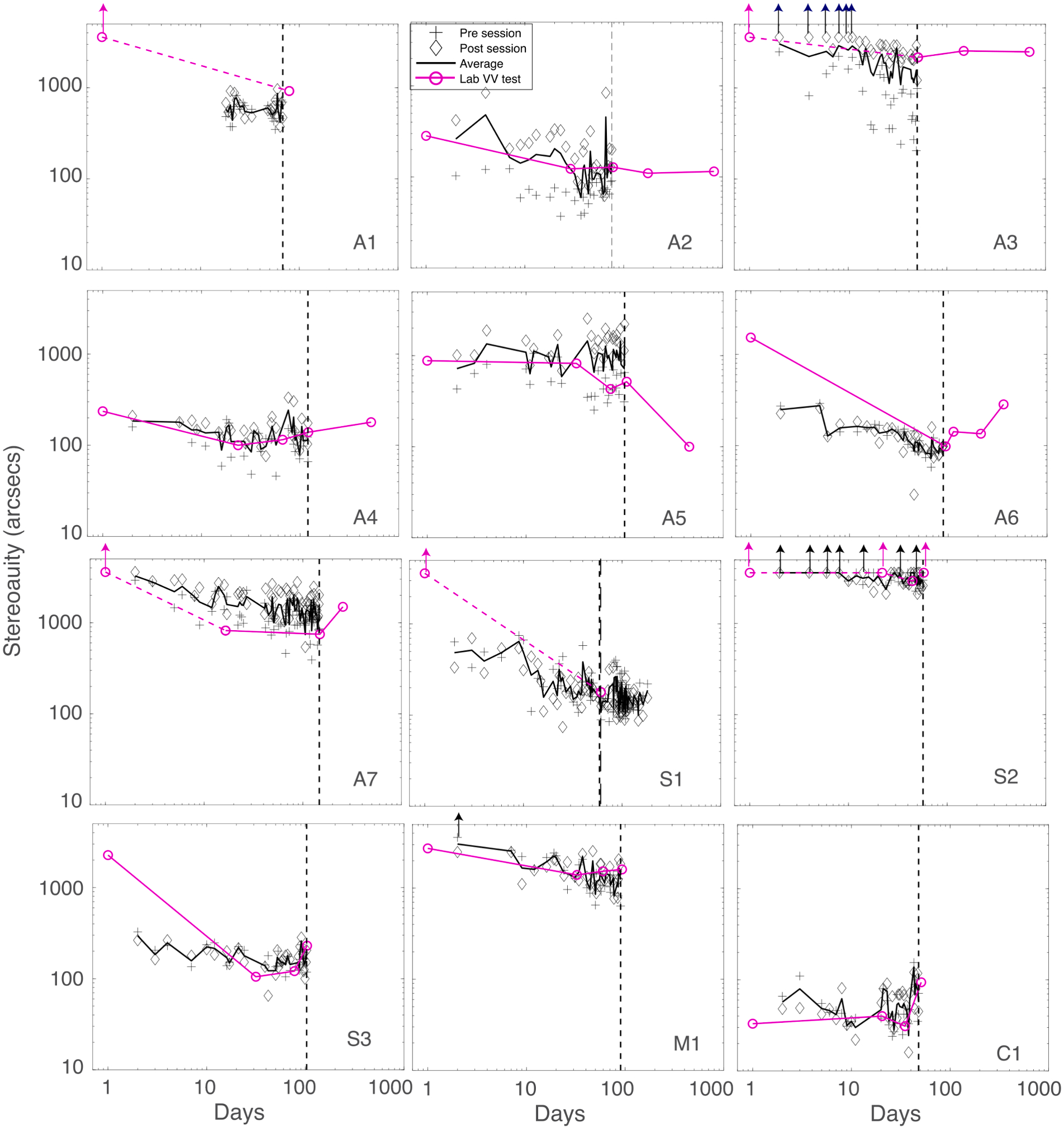
Stereoacuities across the training program measured with the Vivid Vision stereoacuity test. Stereoacuity was assessed with the Vivid Vision stereoacuity test before (gray crosses) and after (gray diamonds) each training session. The black curve shows the average stereoacuity for each training day, calculated from the pre- and post-session measurements. Additional stereoacuity measurements were taken during in-lab visits using the same test (magenta circles). Upward arrows indicate stereoacuity values that exceeded the measurable range of the test. The vertical dashed line represents the last day of training or the completion of 30 training sessions.

Fig. 4 depicts the trajectories of stereoacuity for individual trainees across the binocular vision training program, along with data from a representative control participant (C1). Two additional control participants completed an initial 20-session pilot program conducted at the beginning of the project to evaluate the training procedures. Stereoacuity was assessed before and after each training session using the Vivid Vision stereoacuity test (gray crosses and diamonds, respectively), with the black curve indicating the average stereoacuity across sessions. Additional stereoacuity measurements were obtained during in-lab visits using the same test (magenta circles) when scheduling permitted.

Interestingly, some trainees demonstrated worse stereoacuity after each session (diamonds) compared to their pre-session performance (crosses). This suggests that while game-based therapy may hold potential for improving stereo performance, the observed decline in immediate post-session performance may be attributed to factors such as eye fatigue or the need for more extended neural adaptation.

Trainees A1, A3, A7, S1, and S2 initially had no measurable stereoacuity (upward arrows indicate thresholds exceeding the measurable range of the test). Following training, all but trainee S2 recovered measurable depth perception. Other participants with abnormal stereo vision also showed improvement after training. Follow-up assessments demonstrated that these gains were largely retained over time, although some individuals showed a gradual decline in performance. Extending training beyond 30 h, as in trainees A7 (50 h) and S1 (80 h), did not yield additional benefits.

### 3.2. Binocular disparity thresholds

**Fig. 5.**
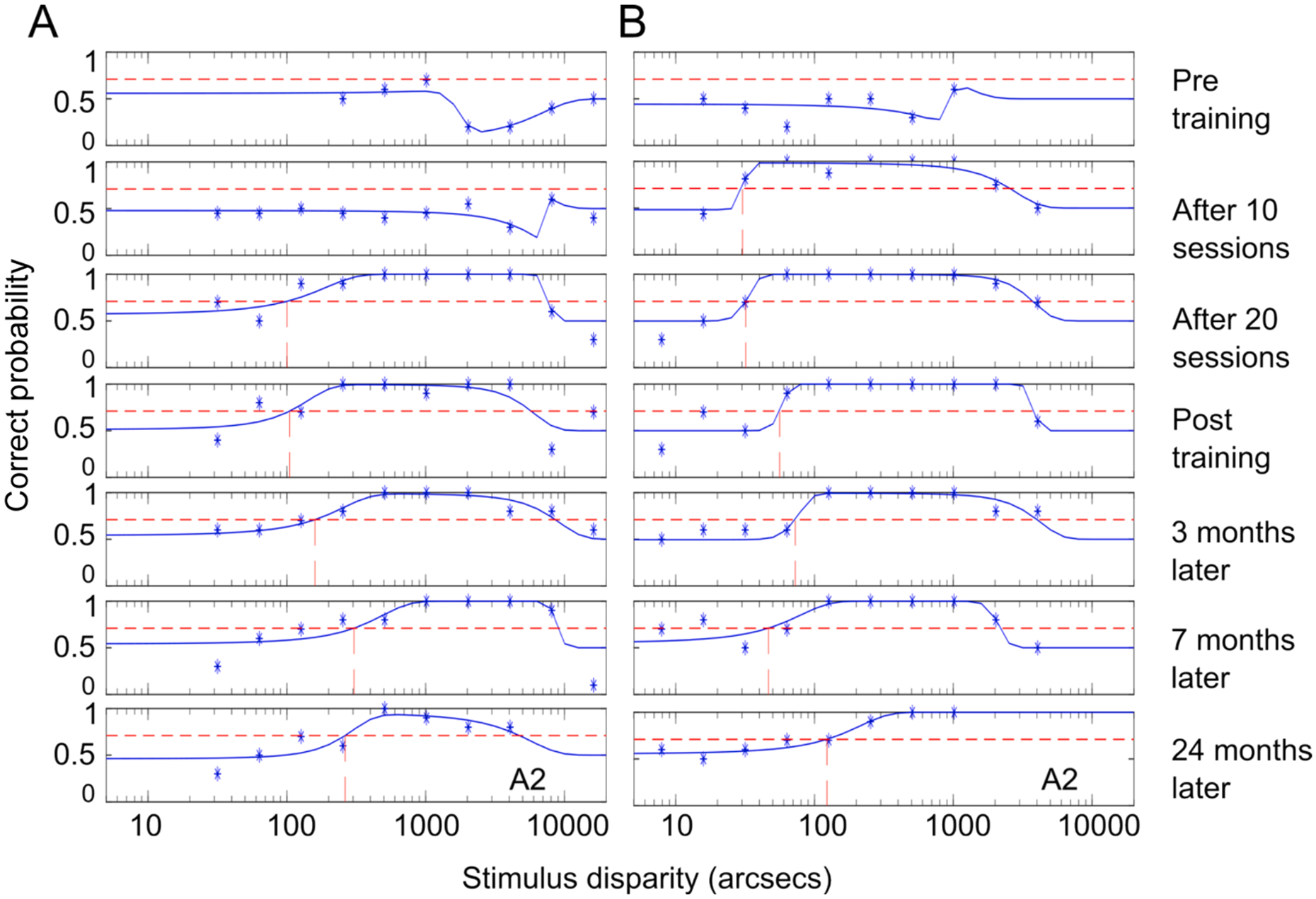
Psychometric functions for trainee A2 measured with either single-plane (Left column) or split-plane (Right column) stimulus. Each curve shows the proportion of correct responses as a function of binocular disparity, fitted with the sum of two cumulative Gaussian functions representing the increasing and decreasing components. The minimum disparity threshold (Dmin) was defined at the 71% correct response level in the increasing component (red dashed lines). Data were collected in blocks of 70–100 trials with 7–10 disparity levels and 10 repetitions.

Psychophysical disparity thresholds were assessed during in-lab visits. Fig. 5 presents psychometric functions for trainee A2, illustrating changes in disparity thresholds across the training program, including pre-training, during-training, post-training, and follow-up assessments. Prior to training, trainee A2 was unable to detect depth either between two depth planes (right column: split-plane condition) or between a single depth plane and the fixation point (left column: single-plane condition), with performance below 71% at all test disparities. After 10 h of training, depth perception between two planes was restored (right), although detection of a single plane relative to fixation remained absent (left). Following 20 h of training, depth perception for a single plane was also recovered. However, once stereopsis emerged, additional training did not further enhance performance; thresholds either stabilized or declined over time. Follow-up assessments indicated a continued decline in the single-plane condition and fluctuations in the split-plane condition. The test conducted 24 months later revealed a marked reduction in depth detection performance for both conditions.

**Fig. 6.**
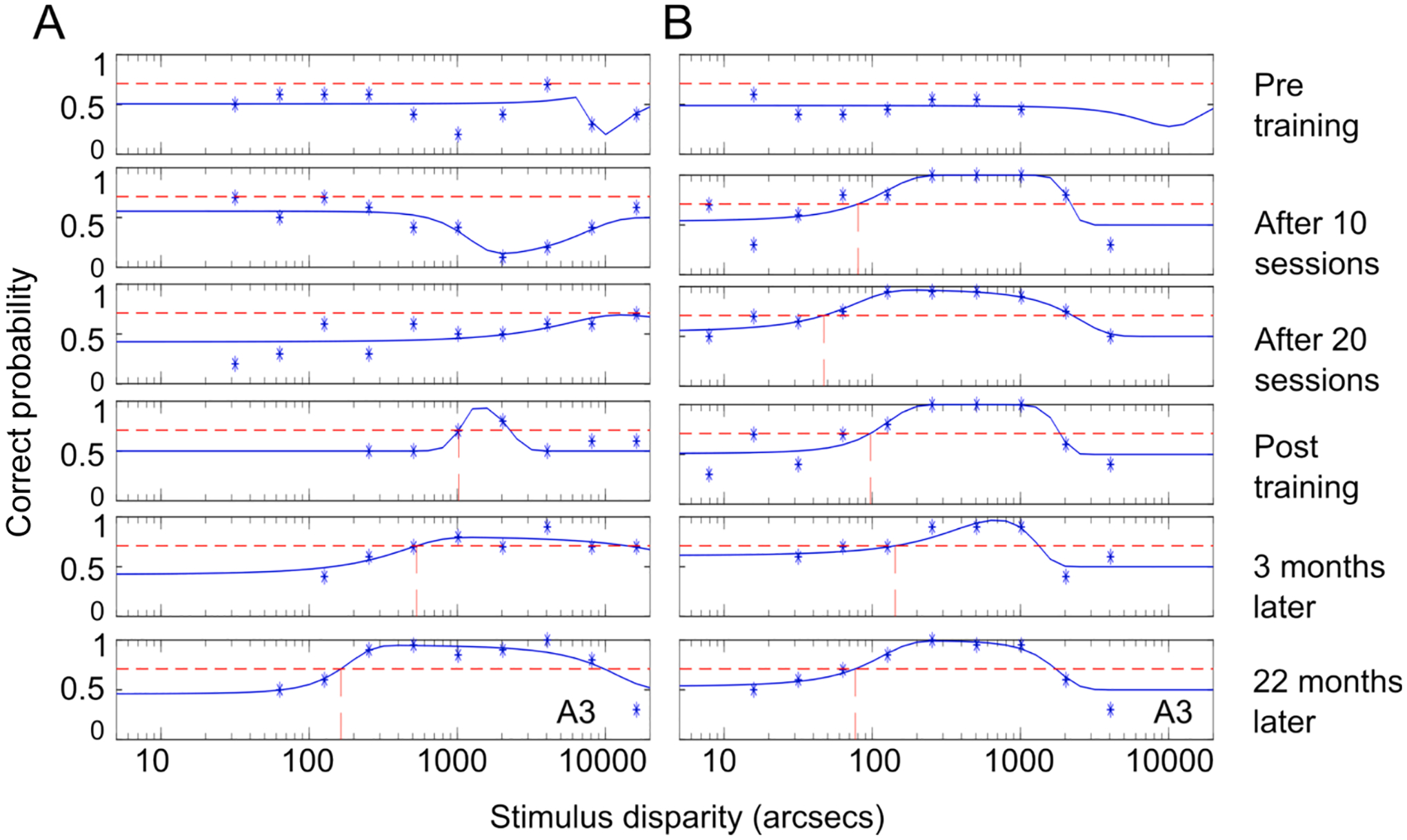
Psychometric functions for trainee A3 measured with either single-plane (Left column) or split-plane (Right column) stimulus.

Fig. 6 shows psychometric functions for trainee A3 across the training program. Similar to trainee A2, before training, trainee A3 was unable to detect depth either between two depth planes (right column) or between a single depth plane and the fixation point (left column). After 10 h of training, depth perception between two planes was restored (right), but detection of a single plane relative to fixation remained absent (left) until the completion of 30 h of training, when single-plane depth perception was recovered. Follow-up assessments revealed a continued improvement in the single-plane condition even without further training, although performance in the split-plane condition fluctuated. The test conducted 22 months later showed a sustained increase in single-plane depth detection, while performance in the split-plane condition remained comparable to that observed after 10 h of training.

**Fig. 7.**
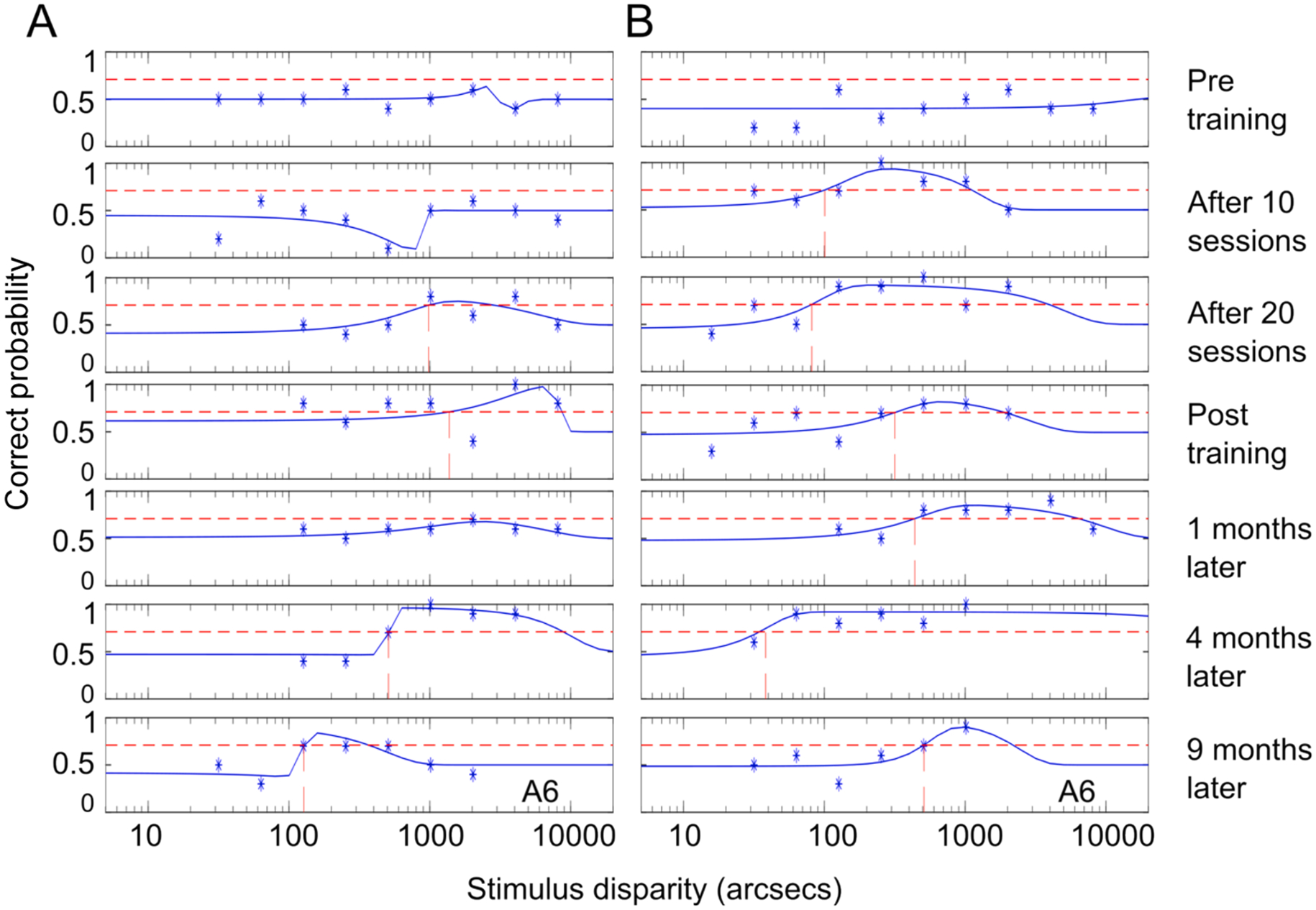
Psychometric functions for trainee A6 measured with either single-plane (Left column) or split-plane (Right column) stimulus.

Fig. 7 presents psychometric functions for trainee A6 across the training program. Similar to trainees A2 and A3, before training, trainee A6 was unable to detect depth either between two depth planes (right column) or between a single depth plane and the fixation point (left column). After 10 h of training, depth perception between two planes was restored (right), although detection of a single plane relative to fixation remained absent (left). Following 20 h of training, depth perception for a single plane was also recovered. Follow-up assessments revealed an initial decline followed by a continued improvement in the single-plane condition, while performance in the split-plane condition fluctuated.

Across the three trainees, stereopsis was absent prior to training, with no measurable depth perception in either the single-plane or split-plane conditions. After approximately 10 h of training, all three trainees demonstrated recovery of depth perception between two planes, whereas single-plane depth detection emerged only after extended training (around 20–30 h). This pattern suggests a sequential restoration of depth perception—first for relative depth between planes, then for absolute depth relative to fixation—based on our limited data. Future studies are needed to confirm this observation.

At first glance, it may seem counterintuitive that these trainees recovered relative (split-plane) disparity perception before single-plane disparity perception, given that detection of the single-plane stimulus relative to fixation might be expected to precede the perception of relative depth (i.e., the disparity difference between two planes). However, the single-plane stimulus included a fixation point that may not have been particularly effective for trainees with poor binocular vision. We suspect that the limited improvement on the single-plane task reflects persistent vergence errors, which would have a smaller impact on relative disparity judgments between the two halves of the split-plane display (see Discussion).

Despite these shared trends, notable individual variability was observed in long-term outcomes: trainee A2 showed stabilization followed by decline, A3 exhibited continued improvement in the single-plane condition even without further training, and A6 displayed an initial decline followed by gradual improvement. Performance in the split-plane condition fluctuated over time for all three trainees. This variability highlights individual differences in neural plasticity and post-training adaptation.

**Fig. 8.**
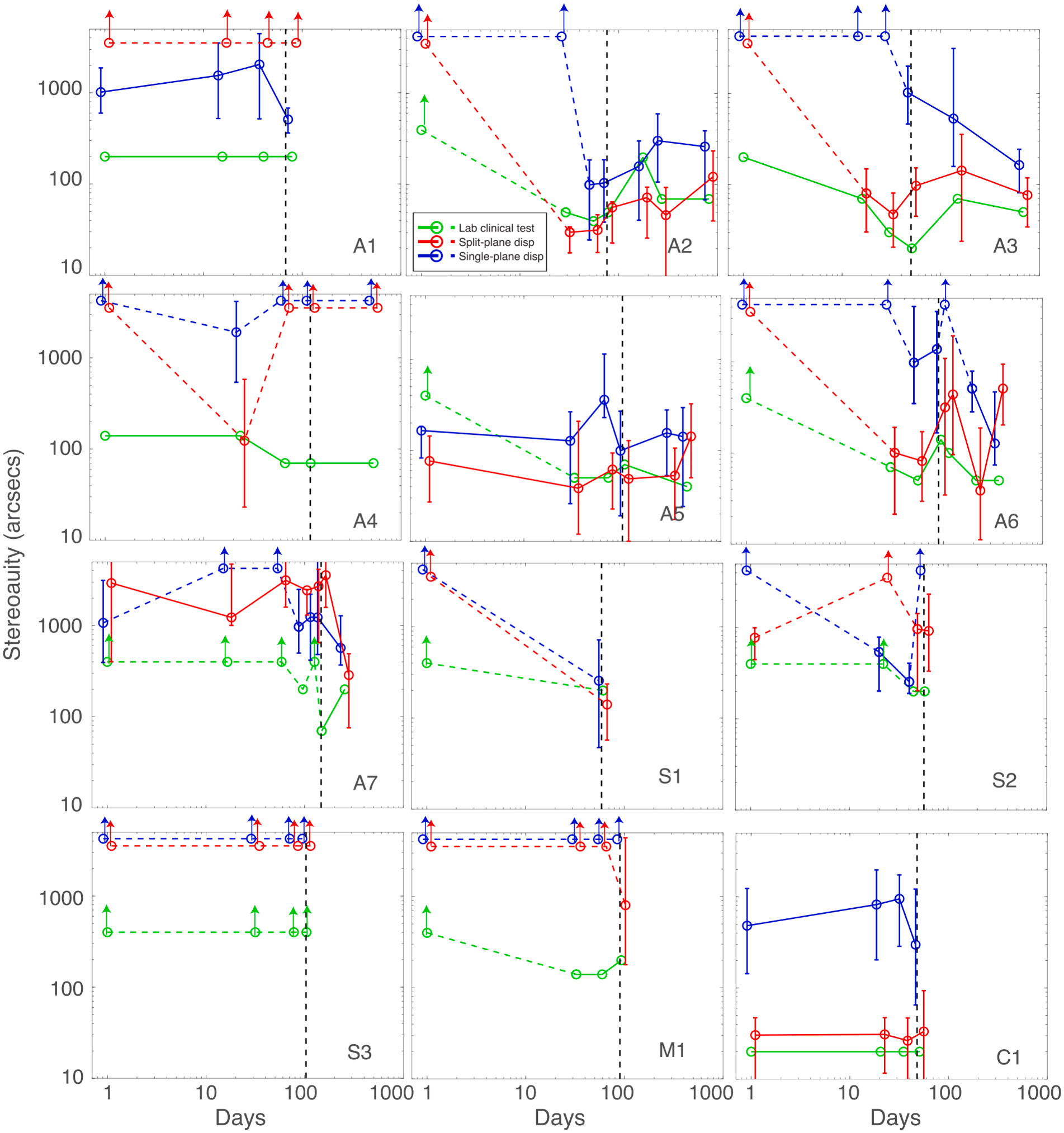
Psychophysical disparity thresholds and clinical stereoacuity measured during in-lab visits. Blue circles indicate single-plane disparity thresholds, red circles indicate split-plane disparity thresholds and green circles indicate clinical stereoacuity. Upward arrows indicate stereoacuity values or disparity thresholds that exceeded the measurable range of the tests. The vertical dashed line represents the last day of training or the completion of 30 training sessions. The error bars represent 95% confidence intervals.

Fig. 8 summarizes psychophysical disparity thresholds for all 11 trainees with abnormal stereopsis and one control participant across the training program. Blue circles denote single-plane disparity thresholds, while red circles represent split-plane disparity thresholds. Similar to trainees A2, A3, and A6, most individuals who initially exhibited poor or unmeasurable stereoacuity showed substantial improvement following training. Clinical circle test results, shown in green, also demonstrated marked gains in most trainees after training. However, there was considerable variability across participants in both the onset of recovery and long-term outcomes, which may reflect differences in ocular conditions, age, or prior exposure to stereo training. For example, trainee A5—who had previously regained stereovision in an earlier training program (Vedamurthy et al. 2015)—displayed a more complex pattern, showing improvements in the Vivid Vision (Fig. 4) and clinical tests but not in the psychophysical measures. Among trainees with strabismic or mixed amblyopia (e.g., S3 and M1), training effects were limited: S3 remained stereoblind in both psychophysical and clinical assessments, whereas M1 recovered stereovision in the single-plane and clinical tests but not in the split-plane test.

**Fig. 9.**
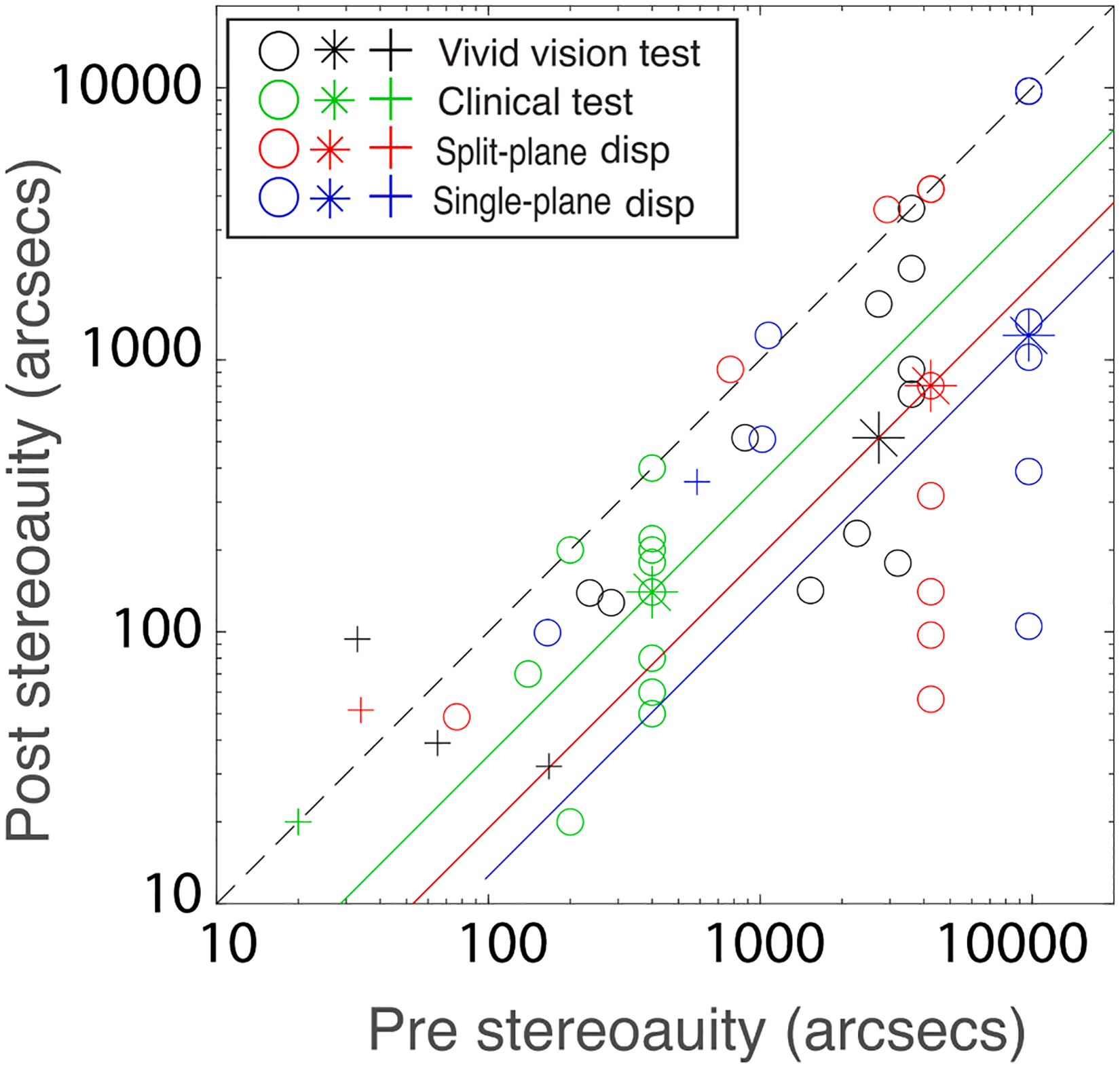
Comparison of pre-training and post-training stereoacuity performance. Circles represent trainees with abnormal binocular vision, and plus symbols denote normal controls. Asterisks indicate the median performance of the abnormal group. For each test, the displacement between the colored median line and the unity (1:1) black dashed line illustrates the magnitude of improvement following training. Ceiling estimates were assigned to unmeasurable thresholds for visualization (see Table 3). Note that the black and red median lines are overlaid. All post-training data were collected at the same time point—after the completion of 30 h of training for each participant, except for controls C2 and C3, who completed 20 h.

Fig. 9 illustrates the effects of training on stereo vision by comparing disparity thresholds before and after training. Prior to training, several trainees (circles) exhibited no measurable stereoacuity, as assessed by the Vivid Vision (black circles), clinical (green circles), and psychophysical (blue and red circles) tests. Plus symbols denote normal controls. For the Vivid Vision and clinical tests, the maximum measurable disparity values were plotted as ceiling estimates, indicating thresholds that exceeded the measurement limit. For the psychophysical tests, ceiling values were set to the average maximum disparity thresholds observed in normal vision, based on our previous study (Ding et al., 2024). Following training, most trainees regained measurable stereoacuity, and those with initially measurable thresholds demonstrated further improvement. A few trainees, however, showed minimal or no improvement, likely due to substantial interocular misalignment.

### 3.3. Statistical analysis of training effects

As shown in Table 3, several trainees improved from unmeasurable to measurable disparity thresholds following training, with no participants showing the reverse pattern. This pattern indicates a general enhancement in disparity sensitivity across tasks.

The McNemar’s test (McNemar, 1947, details see Supplementary Information A) was used to evaluate whether the proportion of participants exhibiting measurable disparity thresholds changed significantly after training. The test examines the null hypothesis that the probability of improvement (from unmeasurable to measurable) equals the probability of decline (from measurable to unmeasurable). Results (see Table 3) revealed a significant increase in the proportion of measurable thresholds for the Circle test (χ^2^ = 5.143, p = 0.0156), with trends toward improvement for the Split-plane (χ^2^ = 3.200, p = 0.0625) and Single-plane (χ^2^ = 2.250, p = 0.1250) tests.

The Wilcoxon signed-rank test (Wilcoxon, 1945, details see Supplementary Information A) was used to assess whether post-training disparity thresholds were significantly lower than pre-training thresholds. Because the thresholds were non-normally distributed and some participants had unmeasurable values, this non-parametric approach provided a more robust alternative to paired t-tests. Ceiling estimates were assigned to unmeasurable thresholds (see Table 3) to enable inclusion in the rank-based analysis. Consistent with McNemar’s results, Wilcoxon tests showed significant post-training reductions in disparity thresholds for the VV (p = 0.0020), Circle (p = 0.0039), and Single-plane (p = 0.0469) tests, as well as a trend toward improvement for the Split-plane test (p = 0.0781) (see Table 3). Together, these results indicate that the training program not only increased the likelihood of measurable stereopsis but also improved the precision of depth discrimination among trainees.

Overall, given the limited dataset, no definitive pattern can be concluded. The training produced only minor and mixed effects on stereoacuity in the normal control (C1) across different measures. Because all three normal controls were pre-screened for a stereoacuity of 20 arcseconds—the best measurable performance on the clinical Randot circle stereotest—little or no improvement was expected or observed on the test (Table 1). Nevertheless, mixed training effects were observed in the other tests among the controls, as shown in Fig. 9 (black plus signs).

Additional results on interocular balance, ocular alignment, equivalent internal noise are provided in the Supplementary Information B. While these findings were more variable across participants and thus less central to our main conclusions, we include them for completeness and transparency, as they may provide useful insights for future studies.

## 4. Discussion

Virtual reality (VR) is emerging as a valuable tool in vision therapy, with successful applications in treating binocular vision disorders such as amblyopia and strabismus (Backus et al. 2018, Coco-Martin et al. 2020, Levi 2023). Its immersive nature makes it more engaging than traditional therapy methods, particularly for children, which can improve compliance with treatment regimens. VR also provides an interactive experience, surpassing methods like patching in effectiveness and appeal, especially for younger patients.

A significant advantage of VR is its ability to provide customized treatment plans tailored to individual patient needs (Backus et al. 2018). In the present study, we utilized the VV prism tuner to estimate the required virtual prism for alignment, applying it individually for each trainee during VV games. Additionally, the difficulty level of the VV games was adjusted based on each trainee’s performance, ensuring personalized and adaptive training. VR systems also facilitate remote therapy, enabling patients to train at home and reducing the frequency of lab visits. These systems allow for progress tracking and provide objective data to inform assessments and treatment adjustments. These features significantly enhanced the efficiency of our study, saving considerable coordination effort and lab time.

Despite these benefits, current VR technology has limitations. Most VR headsets present visual images at a fixed focal distance, creating a conflict between vergence and accommodation. This conflict can cause visual discomfort and reduced performance (Ciuffreda and Thiagarajan 2022, Levi 2022). Moreover, the human visual system is adapted to the statistical properties of disparities typically encountered in natural environments. VR environments, however, often present disparity patterns that deviate from these natural statistics, which can lead to discomfort and diminished visual performance (Aizenman et al. 2023). In the present study, two out of sixteen participants reported sickness or discomfort while using VR, leading to their early withdrawal from the training program. Continued advancements in VR technology and the refinement of disparity presentation are anticipated to address these issues, further enhancing VR's potential in vision therapy.

Following training, all but one of the anisometropic subjects improved to 140 arc sec or better on the clinical Randot stereo test, whereas none of the strabismic subjects had better than 200 arc sec (Table 1).

### 4.1. Considerations of compliance and measurement variability

Trainee compliance is an important factor influencing the effectiveness of home-based training. In this study, compliance was monitored through the Vivid Vision system, which automatically logs training duration, frequency, task completion, and in-game performance. Vivid Vision games are adaptive, providing trial-by-trial feedback and generating performance scores both during and after each session. Although these metrics offer a reliable measure of adherence, they do not fully capture variations in participant engagement or attention, which may contribute to the variability in outcomes observed across participants and tasks. Future protocols could strengthen compliance monitoring by incorporating periodic remote check-ins with researchers or brief self-report measures assessing motivation and engagement.

The use of a stand-alone VR headset without an external display also limited our ability to observe task performance directly. Incorporating an external monitor for in-lab assessments in future studies would allow researchers to identify performance issues early and reduce measurement inconsistencies.

While this study examined multiple components of binocular vision—including stereoacuity, binocular balance, and ocular alignment—the results were not equally consistent across all measures. Stereoacuity exhibited robust and reliable improvements for most participants, aligning with prior evidence of perceptual learning in stereopsis. In contrast, measures such as binocular balance, interocular misalignment, and equivalent noise showed greater variability. To maintain clarity and highlight the most reproducible effects, we present the stereoacuity results in the main text and include the more variable measures in the Supplementary Information. This approach underscores the strongest evidence for VR-based binocular training while preserving full transparency of the dataset and providing a foundation for future methodological refinements.

### 4.2. Mechanistic considerations: Binocular balance and ocular alignment

To evaluate the role of binocular rebalancing in stereo training, we applied a Right-Censored Tobit regression model (Tobin, 1958) (Supplementary Information C) to quantify the association between binocular balance improvement and post-training stereoacuity while controlling for pre-training threshold (Supplementary Information B). This framework was necessary because many participants began training with unmeasurable stereoacuity, producing right-censored data that cannot be analyzed appropriately using standard linear methods. The Tobit model revealed a significant negative relationship between improvements in central-field binocular balance and the latent post-training clinical stereo threshold, indicating that greater contrast balancing in the two eyes is associated with better stereo outcomes. These findings support the idea that sensory reweighting at early binocular stages may play a direct role in enabling improved disparity processing.

In contrast, the present dataset does not allow us to draw firm conclusions about the contribution of ocular misalignment. Although we measured interocular alignment throughout the training period using the VR-based Prism Tuning Test, these measurements exhibited substantial session-to-session variability. Because the stand-alone VR headset did not display the test image on an external monitor, we could not fully monitor task performance, and several measurements were difficult to interpret. As a result, the alignment data were too noisy and inconsistent to support a reliable censored-data regression analysis comparable to that performed for binocular balance. We therefore report the alignment measurements descriptively (Supplementary Information B) but do not attempt to model their relationship with stereo outcomes. This limitation underscores the need for improved alignment-measurement procedures—ideally incorporating built in high resolution eye tracking, and external monitoring, calibration checks, or clinical prism measurements—to allow a more definitive evaluation of the role of motor alignment adjustments in stereo recovery.

Taken together, these results suggest that while sensory rebalancing of binocular contrast appears to contribute meaningfully to stereo improvement, the role of alignment changes remains unresolved. Future work combining precise oculomotor measurements with controlled manipulation of sensory imbalance may help to clarify how these two components interact during binocular rehabilitation and may inform the design of optimized treatment protocols.

### 4.3. Considerations for pediatric VR use

While VR provides a highly immersive and engaging platform for vision training, its use in children requires careful consideration. Off-the-shelf VR headsets often do not fit younger children comfortably, and the vergence-accommodation conflict (VAC) inherent to current VR displays can cause visual strain. Guidelines generally advise limiting VR exposure in children under 12 years of age and keeping sessions relatively short. These practical limitations highlight the need for age-appropriate hardware, careful session planning, and monitoring to ensure safe and effective therapeutic use in pediatric populations.

### 4.4. Local stereopsis training and global stereopsis recovery

Training local stereopsis, which involves matching isolated monocular features between the two eyes, can lead to improvements in global stereopsis (Chopin et al. 2021). However, global stereopsis requires solving a more complex binocular correspondence problem to perceive depth in random-dot stereograms (RDSs). Chopin et al. (2021) proposed a single mechanism for stereopsis with two interacting stages. One stage involves solving the binocular correspondence problem, and the other stage extracts binocular disparities for depth perception.

In the present study, we used home-based VV games and tests, which incorporate monocular features to facilitate binocular fusion, to train and assess local stereopsis. Ten out of eleven participants showed improvements in detecting local stereopsis after training (with the exception of Trainee S2). Notably, six out of nine participants (66.7%) demonstrated improvements in local stereopsis after home-based training, which transferred to improvements in global stereopsis. This global stereopsis recovery was confirmed by in-lab testing using random Gabor patch stereograms (RGPS). This aligns with Chopin et al. (2021), where 60% (3 out of 5) of participants recovered global stereopsis through local stereopsis training. Trainee A5, who initially had both local and global stereopsis, did not exhibit further improvement in global stereopsis after local stereopsis training, despite further improvement in local stereopsis.

In a previous study, we (Ding and Levi, 2021) proposed a unified model for both binocular fusion and depth perception. Similar to the two-stage stereopsis mechanism proposed by Chopin et al. (2021), our model also comprises two stages that operate simultaneously. One stage solves the binocular correspondence problem through a binocular fusion process that minimizes interocular misalignment (phase disparity), while the other extracts position and phase disparity for depth perception. We have now upgraded our model to include both processes of matching monocular features and minimizing interocular misalignment within the fusion mechanism. When stereograms contain monocular features, both processes work together to solve the binocular correspondence problem. However, for stereograms without monocular features (like RDS or RGPS), the model relies solely on minimizing misalignment to solve the correspondence problem. Deficits in minimizing misalignment would impair global stereopsis, while local stereopsis may remain intact due to the presence of a valid feature matching process. Although local stereopsis training may improve performance in both processes, some individuals might rely solely on feature matching for binocular fusion during local stereopsis training, hindering global stereopsis recovery. Therefore, future training protocols should include both local and global stereograms to benefit a wider range of individuals.

### 4.5. Recovery of split-plane disparity detection before single-plane disparity detection

It seems counterintuitive that some individuals might recover split-plane disparity before recovering single-plane disparity, considering the prevailing understanding that relative disparity is calculated from the difference between two absolute disparities. One possible explanation is that while relative disparity detection in the split-plane stimulus improves with training, single-plane disparity might also be improving but remains undetectable due to higher internal disparity noise affecting its detection. Ding et al. (2024) reported that both *global noise* (e.g., vergence noise) and *local noise* contribute to absolute disparity detection. However, for the split-plane stimulus, the global noise is effectively concealed when detecting relative disparity, as it cancels out in the difference between two single-plane disparities, leading to significantly reduced relative disparity thresholds.

Another factor could be the presence of the “absolute disparity anomaly” – a lack of conscious awareness of single-plane disparities (Chopin et al. 2016). This anomaly may be more pronounced in individuals with abnormal binocular vision. In normal vision, absolute disparities are difficult to detect without a fixation point, as there's no reference for depth perception. Adding a fixation point provides this reference, effectively resolving this anomaly (Chopin et al. 2016, Ding et al. 2024). However, for those with abnormal binocular vision, a small fixation point may be less effective as a reference for depth perception. Given that the Gabor patches had a low spatial frequency (0.75 cpd), this could be because that the fixation point and the target Gabor patches were processed through different channels, causing the anomaly to persist when detecting single-plane disparity, even in the presence of a fixation point. Additionally, for amblyopic subjects, the fixation point may only be represented in the non-amblyopic eye dues to suppression of the amblyopic eye, and therefore would not provide a disparity cue.

### 4.6. Limited benefits for Strabismic participants

Our results for interocular misalignment (Supplementary Information B) showed no particular benefits for strabismic participants compared to those without strabismus. This outcome was expected, as strabismus presents unique challenges for binocular alignment and fusion that are not easily overcome with perceptual learning alone. Even when stereoacuity improves in some tasks, persistent ocular misalignment can limit the extent to which binocular integration generalizes across visual functions. These findings are consistent with prior reports that strabismic individuals often require additional interventions—such as surgical alignment, prism correction, or extended rehabilitation—to achieve more robust and stable binocular vision outcomes. Thus, while training may enhance disparity processing in strabismic observers, the lack of specific benefits in ocular alignment underscores the importance of combining training with approaches that directly address motor alignment deficits.

### 4.7. Long-Term effects of VR training for binocular vision

Several studies have reported improvements in stereoacuity after VR training. For example, Godinez et al. (2021) and Portela-Camino et al. (2018) found that participants who played stereoscopic VR games experienced improvement in stereoacuity. Consistent with these studies, the present study also found that VR training improved stereoacuity in adults with abnormal binocular vision due to conditions such as anisometropia, strabismus, and amblyopia. We also found improvement in binocular balance in these individuals.

However, the long-term effects of these improvements are not fully understood. In the present study, while many individuals experience improvements in stereoacuity and binocular balance following VR training, these improvements were not always permanent. Some individuals who regained stereovision during training showed a decline in performance several months after the training period. This suggests that continued practice or “booster” sessions may be needed to maintain the benefits of training. Interestingly, among the four trainees (A2, A3, A5, and A6) with consistent follow-up testing, two (A3 and A6) showed further improvement in the single-plane condition over time after an initial decline during the first few months after stopping training. This may suggest that the brain can continue to adapt and learn from natural binocular cues. However, given the small sample size, firm conclusions cannot be drawn, and further investigation in a larger cohort is needed.

More research is needed to understand the long-term effects of VR training on binocular vision and to optimize training programs for individual needs. Specifically, future studies should focus on: (1) The durability of improvements in stereoacuity and binocular balance after training ends; (2) The optimal duration and frequency of training sessions; (3) The development of standardized protocols and equipment for VR training; (4) whether and how training influences depth perception in the natural environment (i.e., in actual rather than virtual reality).

Declaration of Generative AI and AI-assisted technologies in the writing process

During the preparation of this work the authors used Gemini in order to improve language and readability. After using this tool/service, the authors reviewed and edited the content as needed and takes full responsibility for the content of the publication.

## Supplementary Material

1

## Figures and Tables

**Table 1:** Clinical visual conditions.

Trainees	Age	Sex	Condition	Refractive error		Letter acuity (Snellen)		Stereo (arcsecs)		
				Right eye	Left eye	Right eye	Left eye	Before	After
A1	76	F	Anisometropic	+2.00—1.50×095	+0.50—0.75×090	20/25^−2^	20/20^−2^	200	200
A2	28	F	Anisometropic amblyopia	−0.75	+6.00	20/20	20/125	>400	50
A3	24	F	Anisometropic amblyopia	+0.75—1.25×180	+5.75—5.25×175	20/20	20/50	200	20
A4	28	M	Anisometropic amblyopia	+3.00–4.50×006	+3.50–3.25×172	20/20	20/63	140	70
A5*	61	M	Anisometropic amblyopia	+1.00—1.50×085	+5.75—1.50×077	20/20^−1^	20/50^+2^	>400	70
A6	31	F	Anisometropic amblyopia	+6.5—1.75×165	+1.00	20/40	20/15	>400	140
A7	80	M	Anisometropic amblyopia	+3.0—1.0×035	+1.25—1.25×110	20/40^−2^	20/20^−2^	>400	70
S1	43	F	Strabismic	Plano	Plano	20/25	20/25^−2^	>400	200
S2	38	F	Strabismic	Plano −1.25×012	Plano −0.25×025	20/20	20/25^−2^	>400	200
S3	60	M	Strabismic	+1.25	+0.75	20/50^+1^	20/16	>400	>400
M1	55	M	Mixed amblyopia	+2.50—2.50×163	−2.25—4.00×007	20/20^−2^	20/25	>400	200
C1	24	F	Normal Control	Plano	Plano	20/20	20/20	20	20
C2	20	F	Normal Control	−5.00	−5.00	20/20	20/20	20	20
C3	60	M	Normal Control	−3.50	−4.25—0.25×160	20/20	20/20	20	20

* Previously participated in our previous training program (Vedamurthy et al. 2015).

**Table 2:** Ocular alignment measured with the Vivid Vision Prism Tuning Test.

Trainees	Age	Sex	Condition	BOBI (Δ)		BDBU (Δ)		Cyclotorsion (degree)	
				Before	After	Before	After	Before	After
S1	43	F	Strabismic	0	12.5	0	0	0	0
S2	38	F	Strabismic	100	57	1	2.5	0	0
S3	60	M	Strabismic	6.38	0.59	0.57	0.9	0	0
M1	55	M	Mixed amblyopia	0	7.5	0	0.5	0	0

BOBI: Base-Out Base-In; BDBU: Base-Down Base-UP.

**Table 3:** Results of McNemar’s and Wilcoxon tests comparing pre- and post-training disparity thresholds (N = 11).

Test Condition	Unmeasurable → Measurable	Measurable → Unmeasurable	McNemar’s χ^2^	p (McNemar)	Ceiling (arcsec)	W	p (Wilcoxon)
VV test	3	0	1.333	0.2500	3600	55	0.0020
Circle test	7	0	5.143	0.0156	400	45	0.0039
Split-plane test	5	0	3.200	0.0625	4240	31	0.0781
Single-plane test	4	0	2.250	0.1250	9721	26	0.0469

W: The Wilcoxon statistic, the sum of the signed ranks.

## Appendix A. Supplementary data

Supplementary data to this article can be found online at https://doi.org/10.1016/j.visres.2026.108783.
