# Supplementary material for: Recovery of depth perception in adults with abnormal binocular vision
Source: Vision Res. Author manuscript; Available in PMC 2026 Jul 16. (PMC13374610; doi:10.1016/j.visres.2026.108783)
Supplement: 1 [file NIHMS2191103-supplement-1.docx]

### **Supplemental Information A: Statistical Analyses of Training Effects on Disparity Sensitivity**

To evaluate the effects of perceptual training on disparity sensitivity, two non-parametric tests were used: McNemar’s test for categorical outcomes and the Wilcoxon signed-rank test for continuous paired data. Both are appropriate for small sample sizes and non-normally distributed data.

McNemar’s test (McNemar, 1947) is used to assess whether the proportion of participants exhibiting measurable disparity thresholds significantly changed after training. It tests the null hypothesis that the probability of improvement (from unmeasurable to measurable) equals the probability of decline (from measurable to unmeasurable).

Contingency Table Structure:

|  | Post measurable | Post unmeasurable |
| --- | --- | --- |
| **Pre measurable** | *a* | *c* |
| **Pre unmeasurable** | *b* | *d* |

Here, ‘a’ represents the number of participants who exhibited measurable disparity thresholds both before and after training; ‘b’ represents those who were unmeasurable before but became measurable after training (improved); ‘c’ represents those who were measurable before but became unmeasurable after training (declined); and ‘d’ represents those who remained unmeasurable both before and after training. Only b and c—the discordant pairs—contribute to the McNemar test statistic:

$\chi^{2}\text{=}\frac{\left( b\text{-}c \right)^{2}}{b\text{+}c}$ (A1)

However, because our sample size was small (N = 11), we applied Edwards’ continuity correction (Edwards, 1948) to improve accuracy:

$\chi^{2}\text{=}\frac{\left( \left| b\text{-}c \right|\text{-}1 \right)^{2}}{b\text{+}c}$ (A2)

When b + c < 25, an exact binomial test provides a more accurate p-value (Fagerland et al., 2013). In this case, using MATLAB binomial cumulative distribution function, *binocdf*, the p-value is computed as:

$p\text{=}2\text{*}binocdf\left( min\left( b,c \right),b\text{+}c,0.5 \right)$ (A3)

A significant McNemar test (p < 0.05) indicates that the number of participants who improved after training significantly exceeded those who declined.

The Wilcoxon signed-rank test (Wilcoxon, 1945) was used to assess whether post-training disparity thresholds were significantly lower than pre-training thresholds. Because the disparity thresholds were non-normally distributed and some participants had unmeasurable values, this non-parametric approach provided a more robust alternative to paired t-tests. For participants with unmeasurable thresholds, ceiling estimates were assigned (see Table 2) to enable inclusion in the rank-based comparison.

For each participant, the difference between post-training and pre-training thresholds was computed. The absolute differences were then ranked, and signs were assigned according to the direction of change. The Wilcoxon statistic (W) was calculated as the sum of the signed ranks. Under the null hypothesis of no systematic difference, W follows a known sampling distribution. For small sample sizes (N = 11 in this study), the exact p-value was obtained using the MATLAB function signrank. A significant Wilcoxon test (p < 0.05) indicates that post-training disparity thresholds were significantly lower than pre-training thresholds.

*References*

Edwards, A. L. (1948). *Note on the “correction for continuity” in testing the significance of the difference between correlated proportions.* Psychometrika, 13(3), 185–187.

Fagerland, M. W., Lydersen, S., & Laake, P. (2013). *The McNemar test for binary matched-pairs data: Mid-p and asymptotic are better than exact conditional.* BMC Medical Research Methodology, 13(1), 91.

McNemar, Q. (1947). *Note on the sampling error of the difference between correlated proportions or percentages.* Psychometrika, 12(2), 153–157.

Wilcoxon, F. (1945). *Individual comparisons by ranking methods.* Biometrics Bulletin, 1(6), 80–83.

### **Supplemental Information B: Binocular balance, misalignment, and internal disparity noise**

*Binocular imbalance*

Figure B1 illustrates binocular imbalance—measured using the Vivid Vision Interocular Balance Test—across the training period in the central, parafoveal, and peripheral visual fields. Binocular imbalance is defined as the contrast ratio between the left and right eyes (LE/RE) at the point of binocular rebalance, where a value of 1 indicates perfectly balanced binocular vision. Values >1 reflect right-eye (RE) dominance, whereas values <1 indicate left-eye (LE) dominance.

Pre-training imbalance values are shown on Day 1 (colored circles). Trainees with large initial imbalances (e.g., A3, A6, A7) showed clear improvements and became more binocularly balanced over the course of training. For instance, A3 exhibited a substantial reduction in imbalance during the first 10 days, followed by a rebound later in training. Nevertheless, follow-up testing revealed continued improvement after training ended, with A3 approaching balanced binocular vision (LE/RE ≈ 1) 22 months later—consistent with her long-term recovery in single-plane stereo sensitivity (see Fig. 8).

In contrast, trainees with initially small binocular imbalances (e.g., A1, A4, A5, S1) showed only random day-to-day fluctuations similar to those seen in the normal control C1. Because testing was performed using a stand-alone VR headset without an external monitor display, the experimenter could not fully observe or verify stimulus presentation during testing. As a result, some measurements were difficult to interpret. For example, S3 appeared to show strong RE dominance on Day 1, even though the RE was his amblyopic eye; however, from Day 2 onward, his measurements fluctuated randomly around unity (LE/RE = 1), indicating no systematic imbalance.

*Binocular Balance Improvement*

Binocular balance improvement is defined as the reduction in the absolute deviation from balanced binocular vision after training, i.e., the reduction in ${10}^{\left| {log}_{10}\frac{\text{LE}}{\text{RE}} \right|}$ . Figure B2 summarizes these improvements for trainees with abnormal binocular vision. The vertical distance from each point to the dashed unity line indicates the magnitude of improvement.


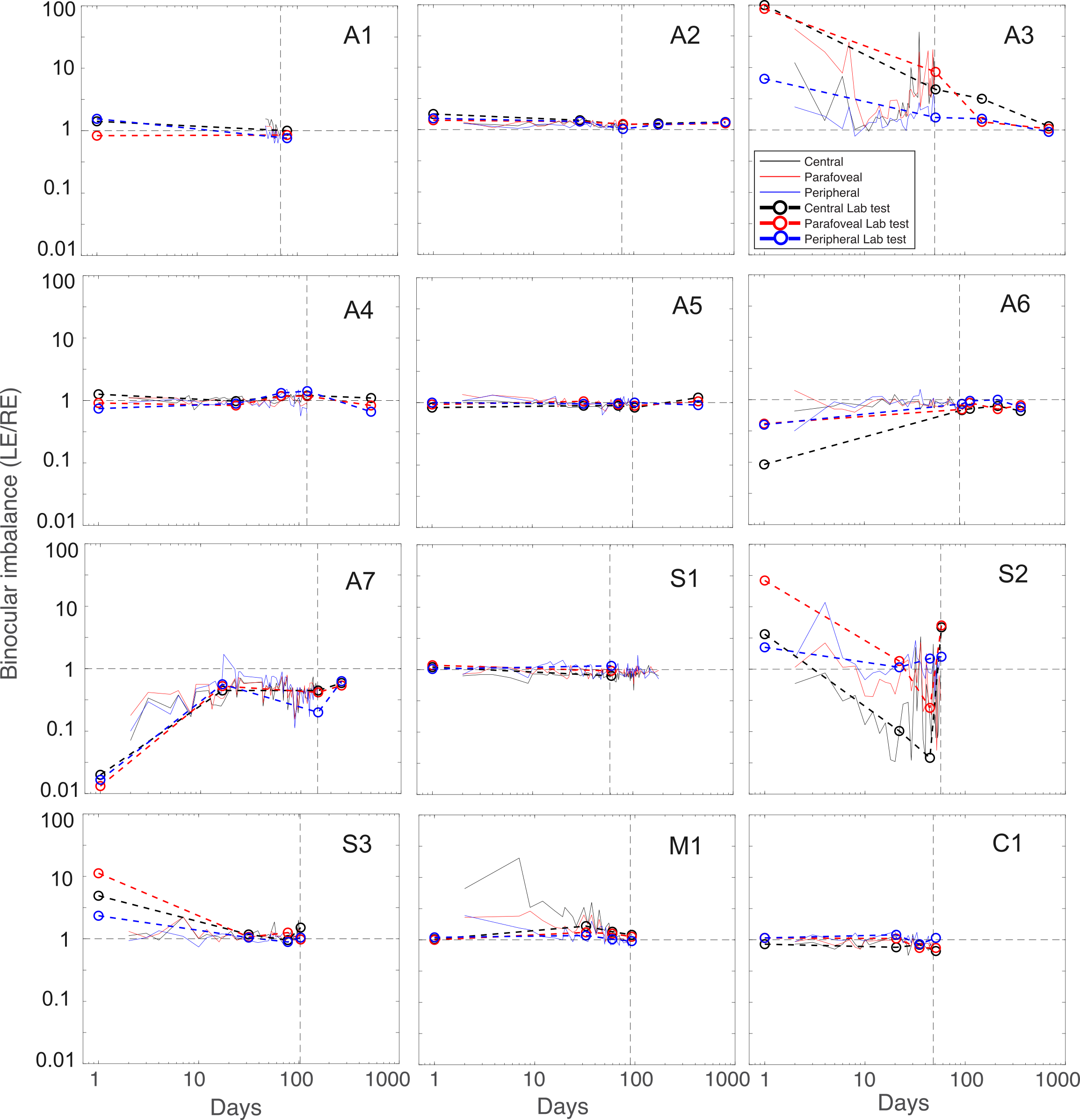


Figure B1. Binocular imbalance measured with the Vivid Vision Interocular Balance Test. Binocular imbalance was measured in the central (black curve), parafoveal (red curve), and peripheral (blue curve) visual areas before each training session. Circles indicate binocular imbalance measured during in-lab visits. The vertical dashed line represents the last day of training or the completion of 30 training sessions. Binocular imbalance is defined as the contrast ratio between the two eyes (LE/RE) at the point of binocular rebalance. Values greater than 1 indicate right-eye dominance, whereas values less than 1 indicate left-eye dominance.

Participants with greater initial binocular imbalance demonstrated the largest improvements across all visual field regions (Fig. B2A). In contrast, those with initially small imbalances showed only minimal training effects, likely reflecting measurement variability. Normal controls (plus symbols) exhibited negligible changes, as expected.


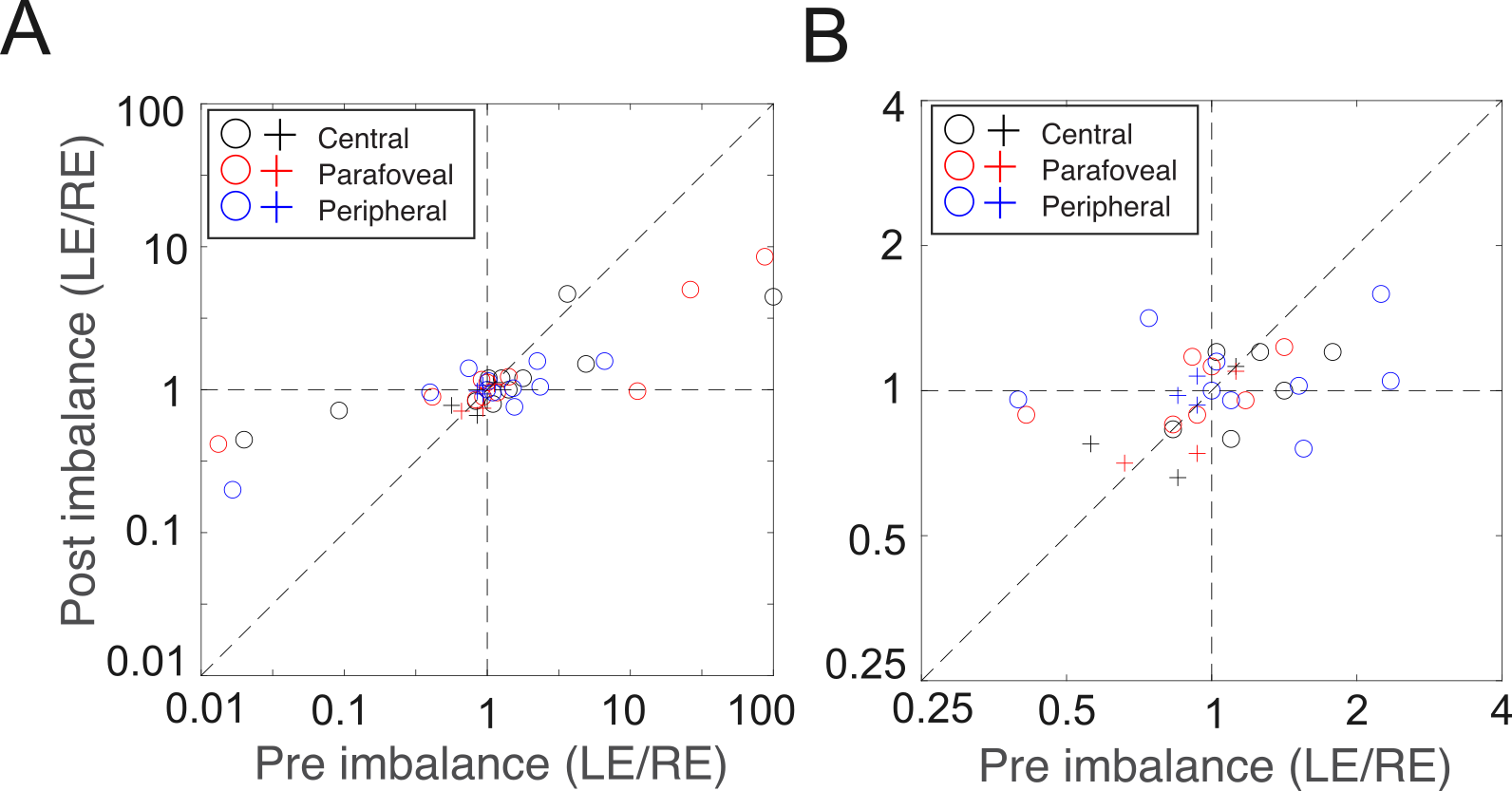


Figure B2. Comparison of pre-training and post-training binocular balance performance. (A) Full-scale plot. (B) Same data plotted with a reduced scale to improve visibility. Circles represent trainees with amblyopia, and plus symbols represent normal controls. All post-training data were collected at the same time point—after completion of 30 hours of training for each trainee, except for controls C2 and C3, who completed 10 hours.

*The relationship between stereoacuity improvement and binocular balance improvement*

To examine the mechanistic relationship between sensory rebalancing and stereoacuity recovery, we quantified changes in clinical stereo thresholds as a function of Binocular Balance Improvement $X$, defined as:

$X\text{=}{10}^{\left| {{log}_{10}\left( \frac{\text{LE}}{\text{RE}} \right)}_{pre} \right|}\text{-}{10}^{\left| {{log}_{10}\left( \frac{\text{LE}}{\text{RE}} \right)}_{post} \right|}$. (B1)

Because many participants began training with unmeasurable clinical stereo thresholds, conventional linear or correlational analyses—restricted only to measurable values—would yield biased estimates. To properly account for these right-censored data (thresholds > 400 arcseconds), we applied a Right-Censored Tobit Regression Model (Tobin, 1958)(Supplement Information C). This framework incorporates the full dataset by using the probability density function (PDF) for measurable thresholds and the cumulative distribution function (CDF) for censored thresholds in the maximum likelihood estimation. All stereo thresholds were log-transformed to stabilize variance and approximate normality, with the censoring point set at 400 arcseconds.

The latent (true but partially unobserved) post-training log-threshold is defined as:

$Y_{post,log,i}^{latent}\text{=}\beta_{0}\text{+}\beta_{1}X_{i}\text{+}\beta_{2}Y_{pre,log,i}\text{+}\epsilon_{i},\text{ }\epsilon_{i}\sim N\left( 0,\sigma^{2} \right)$ (B2)

The observed post-training log-threshold ($Y_{post,log,i}$) is related to the latent log-threshold ($Y_{post,log,i}^{latent}$) by the right-censoring mechanism at the upper log-limit $U_{log}$:

$Y_{post,log,i}\text{=}\left\{ \begin{aligned} Y_{post,log,i}^{latent}\text{ if }Y_{post,log,i}^{latent}\text{<}U_{log}\text{ }\left( Observed \right) \\ U_{log}\text{ if }Y_{post,log,i}^{latent}\geq U_{log}\text{ }\left( Censored \right) \end{aligned} \right.$ (B3)

Where $X_{i}$ is the quantitative measure of Binocular Balance Improvement, given by Eq. B1, and $Y_{pre,log,i}$ is the Pre-Training Log-Threshold (using the observed log-value, but $Y_{pre,log}\text{=}U_{log}$ for unmeasurable pre-training thresholds).

Figure B3 summarizes the clinical stereo-threshold data, the corresponding central-field binocular balance measurements, and the resulting model predictions. Figure B3A plots post-training stereo thresholds against pre-training thresholds on the log scale, with each point color-coded by the magnitude of Binocular Balance Improvement in the central visual field. Figure B3B shows the predicted latent post-training stereo threshold as a function of central-field binocular balance improvement. The solid blue line represents the predicted recovery curve for participants who began at the censoring limit (400 arcseconds), whereas the dashed red line depicts predictions for participants with the average pre-training threshold.

The Right-Censored Tobit model, controlling for pre-training threshold, revealed a significant negative association between central-field Binocular Balance Improvement and the latent post-training stereo threshold (see Slope $\beta_{1}$ in Table B1). Thus, greater improvement in central binocular balance predicts a lower (better) latent stereo threshold following training. In contrast, the pre-training stereo threshold was not a statistically significant fixed-effect predictor.

Table B1. Tobit model results for clinical stereo thresholds

| Parameter | Estimate | SE | T_stat | P_value |
| --- | --- | --- | --- | --- |
| Intercept $\beta_{0}$ | 0.905 | 1.380 | 0.655 | 0.512 |
| Slope $\beta_{1}$ | -0.0075 | 0.003 | -2.479 | 0.013 |
| Slope $\beta_{2}$ | 0.501 | 0.545 | 0.919 | 0.358 |
| Std Dev $\sigma$ | 0.279 | 0.064 | 4.353 | 1.35e-05 |


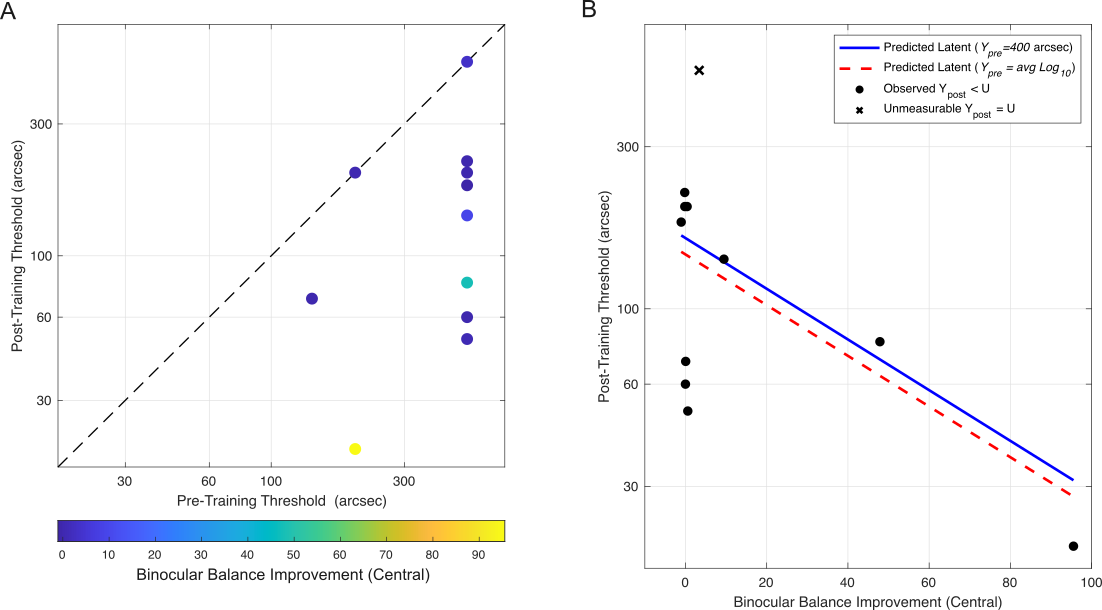
Figure B3. **Stereo performance improvement on clinical circle test.** **(A) Comparison of Pre- and Post-Training Stereo Thresholds.** Post-training thresholds are plotted against pre-training thresholds in log units. Data points are color-coded by the magnitude of the central-field Binocular Balance Improvement predictor. The maximum measurable threshold is U = 400 arcsec; censored data points (unmeasurable) are slightly offset above the ceiling for visualization. **(B) Right-Censored Tobit Model Predictions for Latent Threshold.** The predicted latent post-training stereo threshold is shown as a function of the central-field Binocular Balance Improvement predictor. The ***solid blue line*** represents the predicted threshold for participants who began at the censoring limit (400 arcsec), and the ***dashed red line*** represents the prediction for participants at the average observed pre-training threshold in the log unit. Raw data points are overlaid.

However, improvements in the Vivid Vision, split-plane, and single-plane stereo tests were not significantly associated with central-field binocular balance improvement, as indicated by the Right-Censored Tobit model analyses (Figs. B4–B6). Likewise, improvements across all stereo tests showed no significant association with binocular balance improvements in the parafoveal or peripheral visual fields.


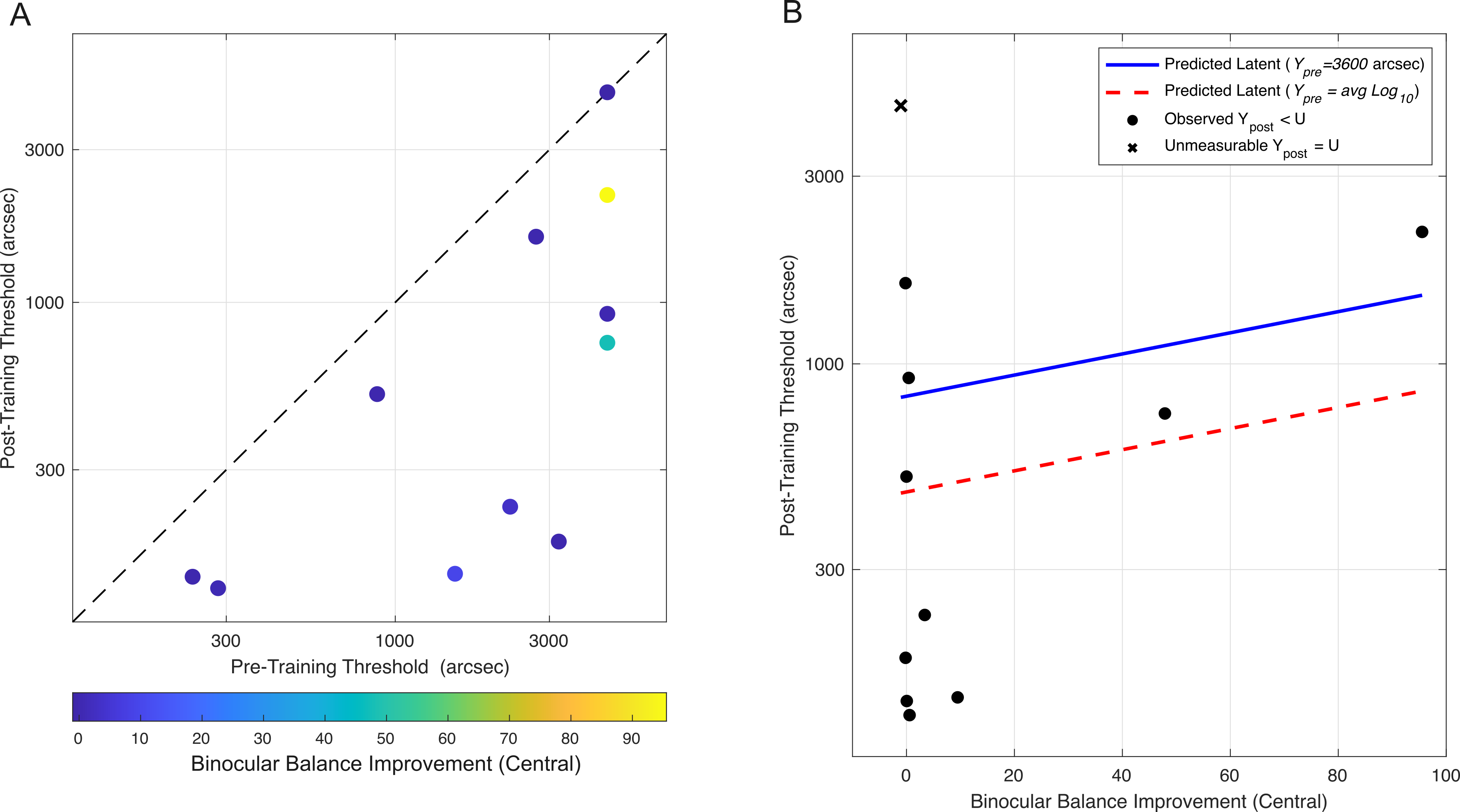
Figure B4. Stereo performance improvement on the Vivid Vision stereo test. The maximum measurable threshold is U = 3600 arcseconds; censored data points (unmeasurable) are slightly offset above the ceiling for visualization. The estimated slope ($\beta_{1}$ ) for the association between central-field binocular balance improvement and latent post-training stereo threshold is 0.0027 (p = 0.546).


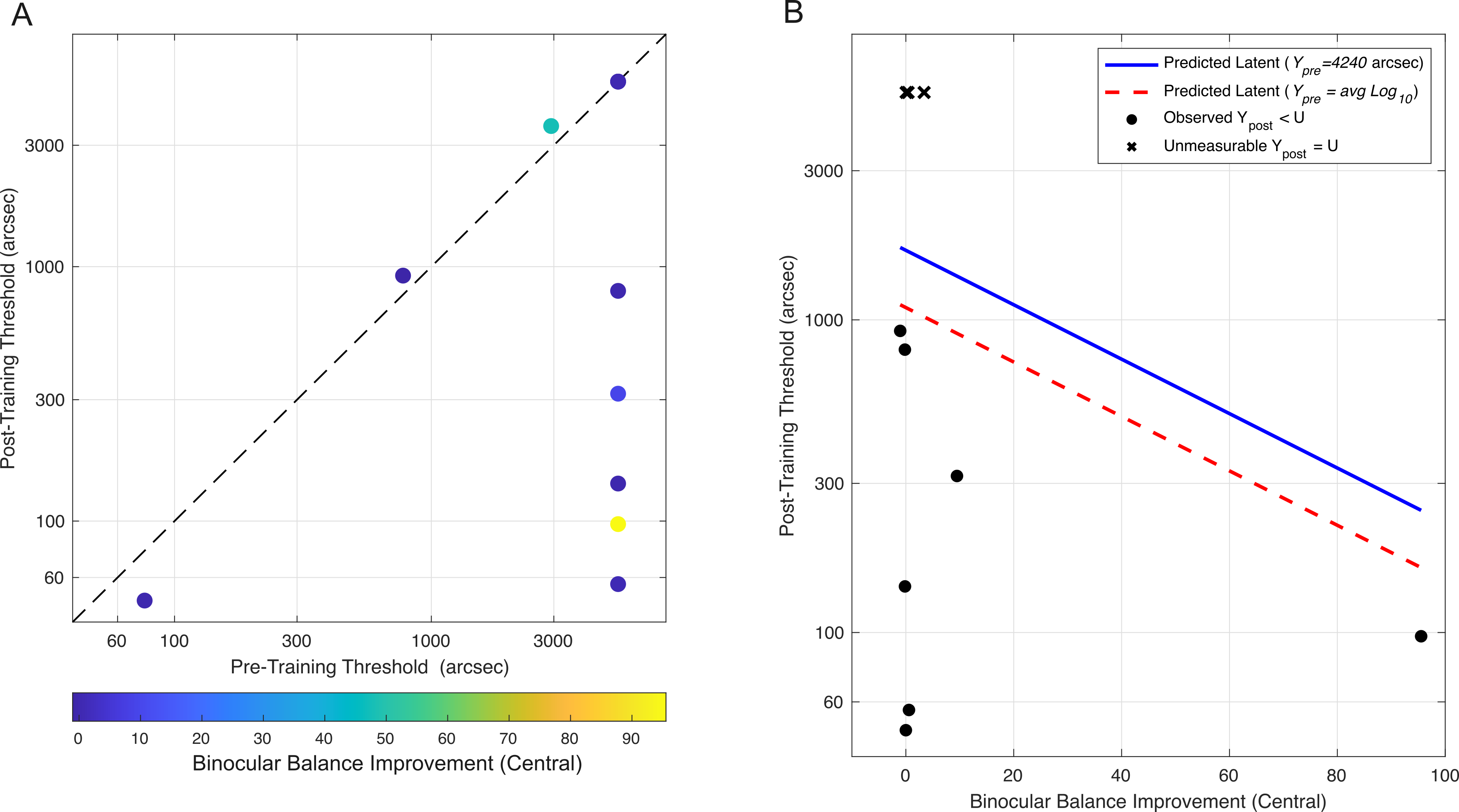
Figure B5. **Stereo performance improvement on the split-plane stereo test.** The maximum measurable threshold is U = 4240 arcsec; censored data points (unmeasurable) are slightly offset above the ceiling for visualization. The estimated slope ($\beta_{1}$ ) for the association between central-field binocular balance improvement and latent post-training stereo threshold is -0.0087 (p
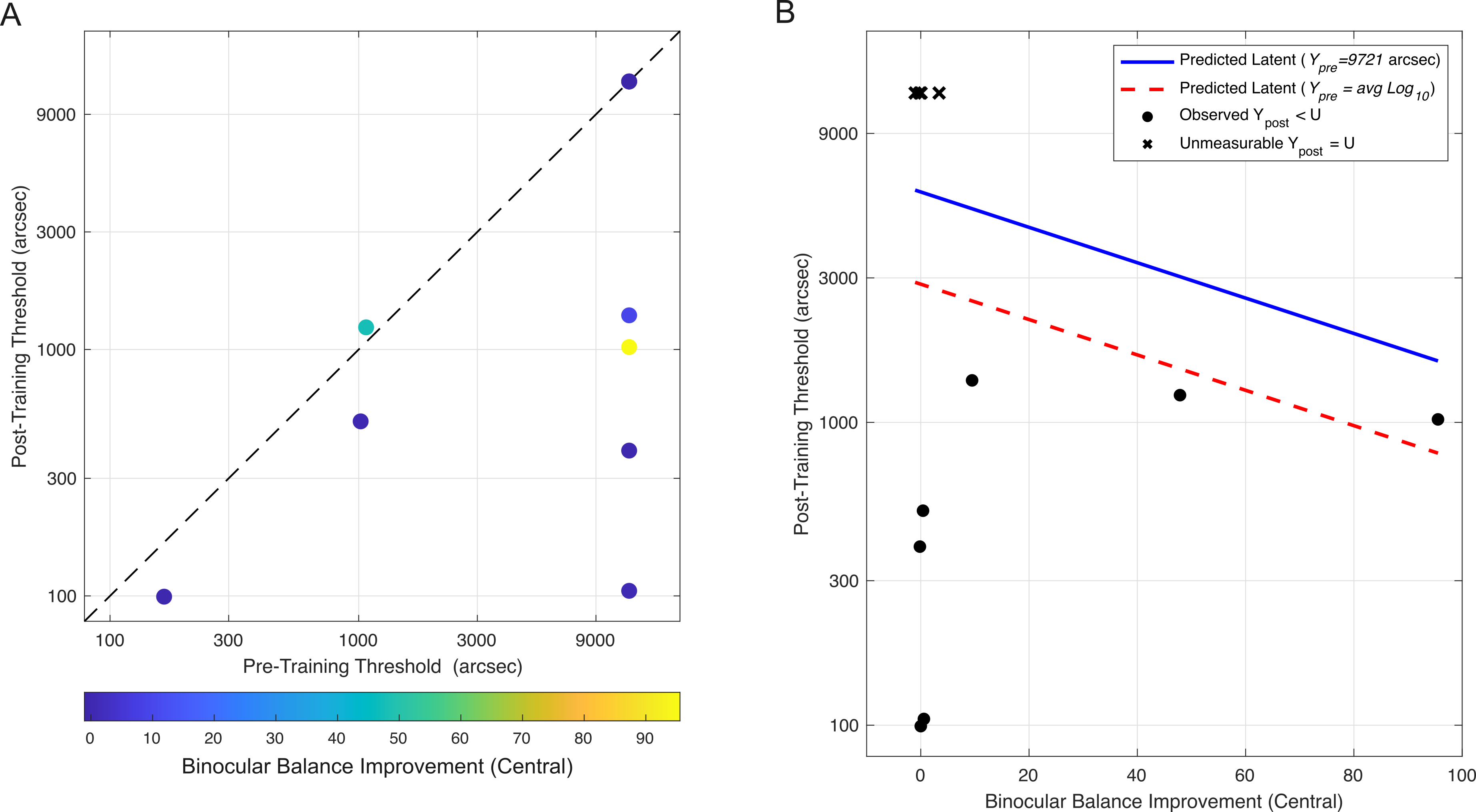
 = 0.351).

Figure B6. **Stereo performance improvement on the single-plane stereo test. The maximum measurable threshold is U = 9721 arcsec; censored data points (unmeasurable) are slightly offset above the ceiling for visualization. The estimated slope (**$\beta_{1}$ **) for the association between central-field binocular balance improvement and latent post-training stereo threshold is -0.0058 (p = 0.549).**

*Interocular misalignment*

Figure B7 illustrates interocular misalignment throughout the training program, quantified by the amount of virtual prism required for alignment using the Vivid Vision Prism Tuning Test. A positive misalignment value indicates the need for a Base-Out prism for horizontal correction or a Base-Down prism for vertical correction. Conversely, a negative misalignment value indicates the need for a Base-In prism for horizontal correction or a Base-Up prism for vertical correction. As with the binocular imbalance measurements, the use of a stand-alone VR headset without displaying the test image on an external monitor limited our ability to fully monitor task performance. Consequently, some alignment measurements were difficult to interpret. For example, A1 appears to begin with a 30–prism-diopter horizontal deviation despite being an anisometropic amblyope, and A3 shows a similar inconsistency. Conversely, some strabismic or mixed-type participants show no detectable misalignment in the pre-training test but exhibit fluctuations during training (e.g., S1, M1). In addition, the measurement noise of this VR-based alignment task appears to be substantial: day-to-day variability was often large and not accompanied by corresponding changes in binocular balance or stereoacuity. To avoid over-interpretation, we present these measurements descriptively and do not draw conclusions beyond what the raw data support.


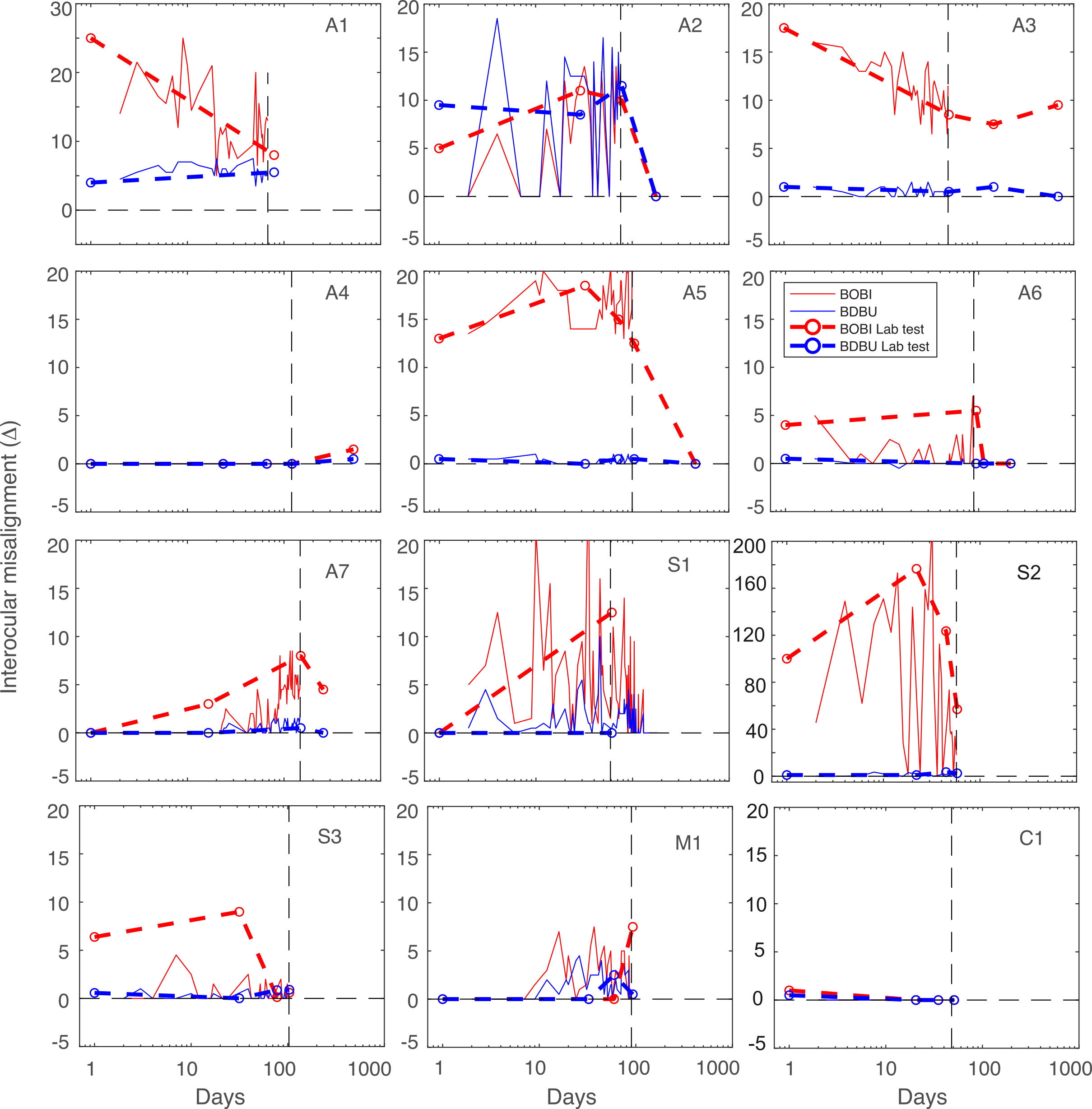
Figure B7. Interocular misalignment throughout the training program measured with the Vivid Vision Prism Tuning Test. The virtual prism required for alignment is measured as interocular misalignment before each session. The red curve represents horizontal misalignment (Base-Out positive, Base-In negative), while the blue curve represents vertical misalignment (Base-Down positive, Base-Up negative). Circles denote misalignments measured during in-lab visits. The vertical dashed line indicates the last day of training or after 30 training sessions.


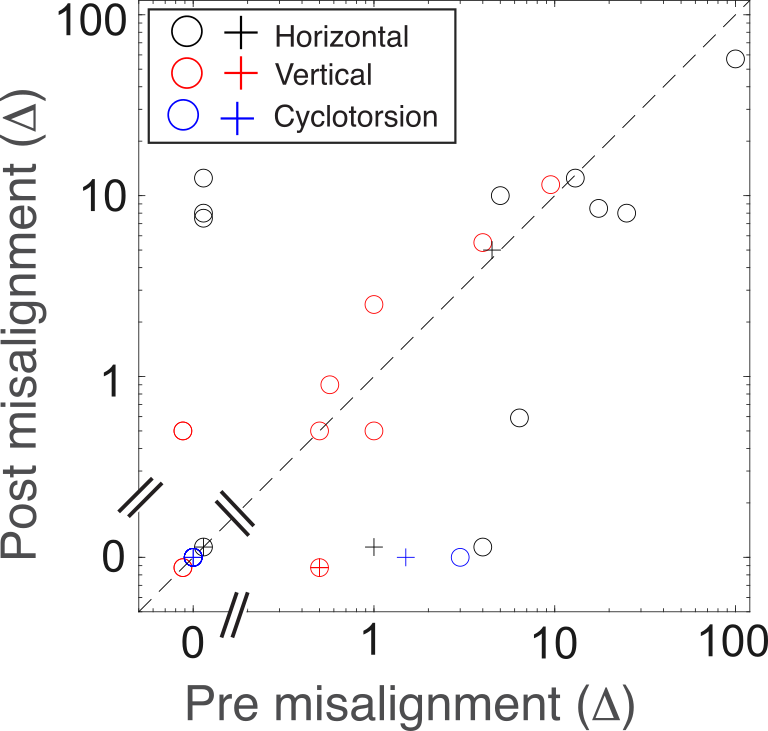


Figure B8. Comparison of pre-training and post-training interocular misalignment. Circles represent trainees with amblyopia, and plus symbols represent normal controls. Small horizontal and vertical offsets were applied to points at zero to reduce overlap and improve visibility. All post-training data were collected at the same time point—after completion of 30 hours of training for each trainee, except for controls C2 and C3, who completed 20 hours.

*Internal disparity noise*

In addition to clinical visual acuity and stereoacuity assessments, in-lab tests included measurements of equivalent internal disparity noise and detection efficiency for both single- and split-plane disparity. These measures were obtained using an equivalent-noise paradigm (Ding, Lu & Levi, 2024). External disparity noise was added to the stimuli, and disparity thresholds were measured as a function of the external noise standard deviation (Figure B9). As expected, thresholds remained relatively constant at low external noise levels but increased proportionally with external noise at higher noise levels. The transition point—marked by a colored vertical bar on the x-axis—provides an estimate of the standard deviation of the equivalent internal disparity noise. The smooth colored curves represent the best-fitting model functions, parameterized by two quantities: equivalent internal disparity noise and detection efficiency (Ding, Lu & Levi, 2024). Equivalent internal disparity noise is the level of internal noise within the visual system that limits disparity processing. It reflects the variability or uncertainty in the observer’s binocular disparity signals. Efficiency quantifies how effectively the observer uses the available disparity information.

The estimated internal noise values are shown by the colored bars at the bottom of each panel: blue bars correspond to single-plane disparity detection, and red bars correspond to split-plane disparity detection. Short, wide bars indicate individual trainees’ internal noise estimates, whereas long, narrow bars denote the average noise levels from three normal controls reported in Ding, Lu et al. (2024).

However, this task was very challenging for most trainees with abnormal binocular vision. Although it was administered at every in-lab visit, only a subset of trainees were able to produce meaningful data after regaining measurable stereovision at different time points, as shown in Figure B8. Trainee A5 (the first row) had previously participated in our previous training program (Vedamurthy, Nahum et al. 2015) and recovered stereo vision before entering the current program. While some improvement was observed during training, his performance returned to pre-training levels and remained stable. A2 (the second row) and A3 (the third row) both lacked stereovision before entering the program. After 10 hours of training, both regained split-plane disparity perception comparable to normal controls (dashed lines). However, their single-plane disparity threshold remained unmeasurable, potentially due to persistent vergence errors, which have a greater impact on the visual system’s ability to measure disparity for small isolated features (the fixation mark) than on large display regions. A2 regained single-plane disparity detection after 20 hours, while A3 did so after 30 hours, although with poor performance and declining relative disparity detection over time. However, at the first follow-up (3 months later), A3's performance stabilized for both single- and split-plane disparity, albeit at low levels, while A2's performance declined in both. At 7 months, A2's single-plane disparity detection further decreased, with split-plane remaining stable. A3 was unavailable at 7 months and completed the second follow-up at 22 months. Interestingly, after almost two years, her split-plane performance remained stable, and her single-plane performance had even improved considerably. A similar pattern, with initial recovery during training, subsequent performance decline shortly after stopping training, and then further recovery long after training, was also observed in A6 (last row). This suggests that the recovery of depth perception is a dynamic process, during which a temporary loss in performance may occur, and performance requires more time to become consolidated after training.

Trainees showed varying degrees of improvement in stereo vision after training. Some regained depth perception comparable to normal controls, while others showed limited or unstable improvements. Some trainees struggled with perceiving single-plane depth but could perceive split-plane depth. This may be due to persistent issues with eye coordination (vergence errors). The recovery process was dynamic, with some trainees experiencing initial gains followed by declines in performance after stopping training. However, some showed further improvement over time, even without continued training. This suggests that the brain continues to adapt and learn even after the training period ends. This study highlights the potential for recovering stereo vision through training. However, the recovery process is complex and varies between individuals. Further research is needed to understand the factors that influence successful recovery and to optimize training programs for different needs.


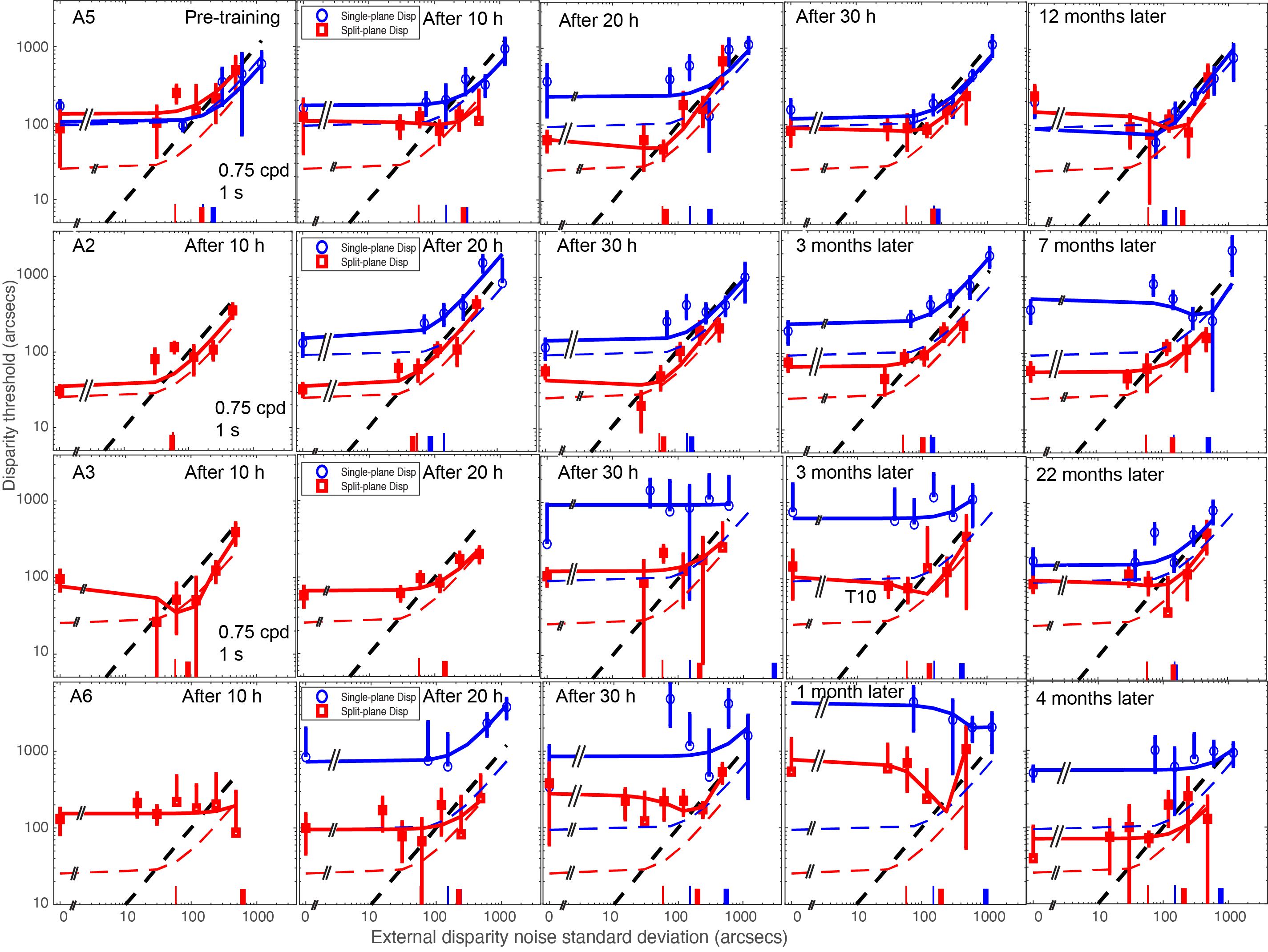


Figure B9. Disparity thresholds as a function of external disparity noise standard deviation across training programs, from pre-training to follow-up tests. Solid lines represent amblyopic trainees, and dashed lines (overlaid in some panels) represent normal controls. Short colored bars indicate equivalent internal noise in amblyopic trainees, and long slim colored bars indicate equivalent noise in normal controls. The error bars represent standard errors. Panels show performance on the single-plane and split-plane disparity tasks for four subjects (A3, A5, A6, A7) measured at different time points during training. Many participants initially failed the task due to very poor stereoacuity, which is why pre-training data are missing for some subjects. Some subjects who initially failed regained measurable performance after approximately 10 hours of individualized training. Control data (black plus symbols) represent the average performance of three normal-sighted participants from a previous study (Ding, Lu & Levi, 2024) and are included in all panels as a normative benchmark; in some panels, the control data are overlaid on participant traces. These data illustrate both the effects of training and variability across participants.

As shown in Figure B10, internal disparity noise, measured through equivalent noise analysis (Ding, Lu et al. 2024), fluctuates over time. When initial internal noise is low at recovery, it tends to increase over time. Conversely, when initial internal noise is high at recovery, it tends to decrease. For trainees with initial stereopsis—either one whose stereopsis was recovered in a previous study (Vedamurthy, Nahum et al. 2015)(A5) or a control trainee (C1)—internal noise fluctuated around an average level over time. We hypothesize that, for individuals with abnormal binocular vision, the level of initial internal noise at recovery may vary due to differences in the strength of surround interactions. Surround interactions are essential for visual processing in complex environments, including contrast perception, edge detection, segmentation, and contour integration. Previous studies have shown that surrounds at different depths influence depth perception in individuals with normal vision (Mitchison and McKee 1985, Westheimer and Levi 1987, Ocansey, Osuobeni et al. 2019).  Normal surround interactions may play a critical role in depth perception. Abnormal surround interactions—either weaker or stronger than normal—may lead to impaired depth perception. Interestingly, by the end of the training program, the final internal noise levels of relative disparity for four anisometropic trainees converged to a similar level, approximately three times higher than normal controls (indicated by the red horizontal dashed line). This was observed regardless of whether the initial noise at recovery was lower (A2 and A3) or higher (A6), or if the trainee had pre-training stereopsis (A5). The strabismic trainee (S1) also exhibited a similar internal noise level after training. However, due to the limited data, this observation should be considered preliminary and requires further confirmation.

Given that internal noise can originate from various stages in the signal processing pathway—such as input, detection, and decision stages—it is plausible that higher initial internal noise may result from greater decision uncertainty at the time of recovery. As decision uncertainty decreases over training, overall internal noise is reduced, leading to improved depth perception.


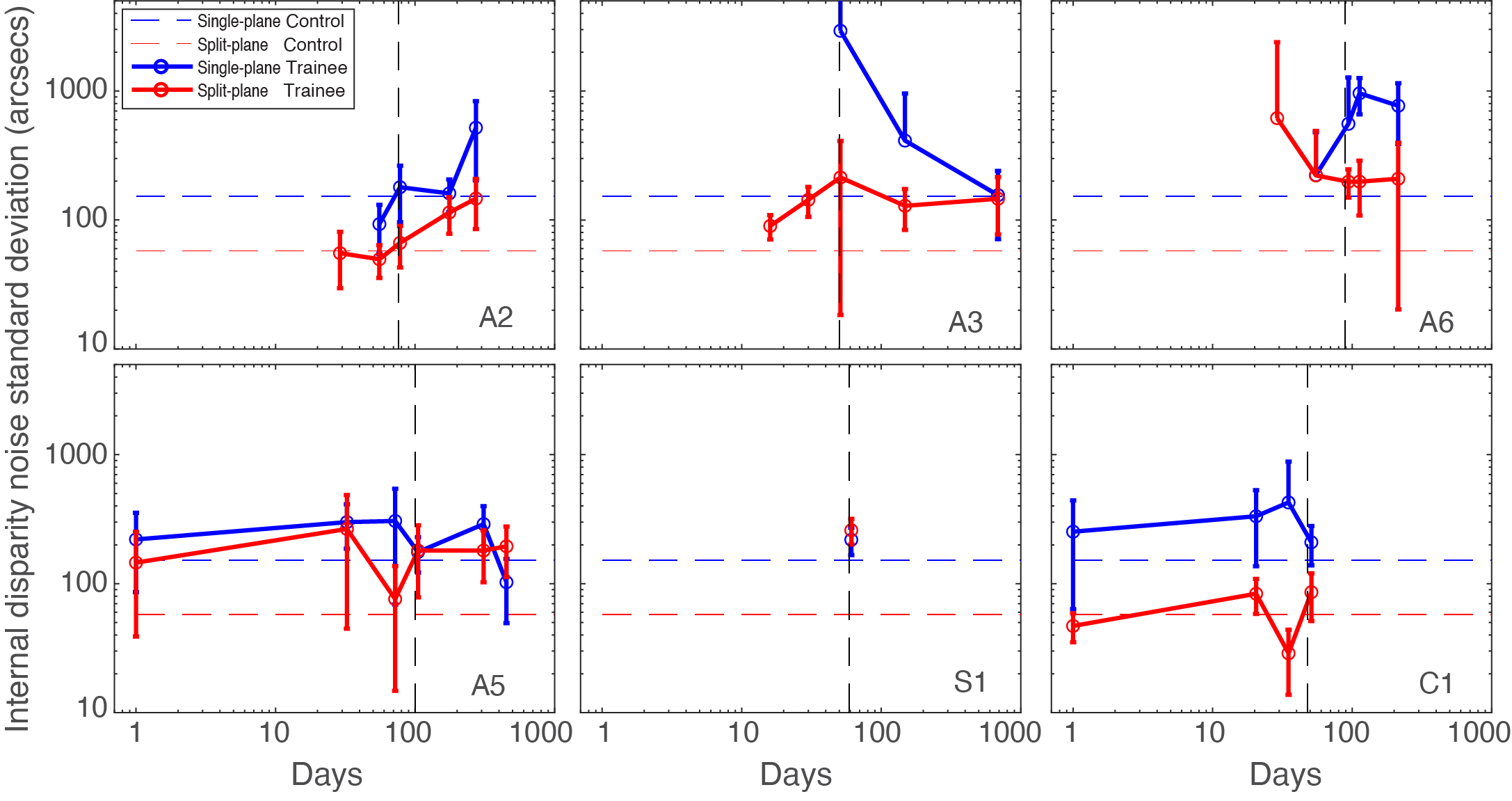


Figure B10. The standard deviation of internal disparity noise across the training program. Internal disparity noise was evaluated through equivalent noise analysis (Ding, Lu et al. 2024). The horizontal lines indicate the averaged noise levels of three normal controls from Ding, Lu et al. (2024). The vertical dashed line represents the last day of training or the completion of 30 training sessions. The error bars represent standard errors.

*References*

Ding, J., H. H. Lu and D. M. Levi (2024). "Absolute and relative disparity mechanisms revealed by an equivalent noise analysis." Sci Rep **14**(1): 6863.

Mitchison, G. J. and S. P. McKee (1985). "Interpolation in stereoscopic matching." Nature 315(6018): 402-404.

Ocansey, S., E. Osuobeni and J. Siderov (2019). "Lateral interference, effects of flankers and reference bar configuration on foveal depth discrimination thresholds." Vision Res 156: 96-104.

Tobin, J. (1958). Estimation of relationships for limited dependent variables. Econometrica: Journal of the Econometric Society, 24-36.

Vedamurthy, I., M. Nahum, S. J. Huang, F. Zheng, J. Bayliss, D. Bavelier and D. M. Levi (2015). "A dichoptic custom-made action video game as a treatment for adult amblyopia." Vision Res 114: 173-187.

Westheimer, G. and D. M. Levi (1987). "Depth attraction and repulsion of disparate foveal stimuli." Vision Research 27(8): 1361-1368.

**Supplemental Information C: Right-Censored Tobit Model**

*Rationale for Using the Right-Censored Tobit Model*

Standard linear regression assumes that the dependent variable is continuous and fully observed. However, our stereo threshold data violates this assumption, requiring specialized statistical treatment. Trainees with abnormal binocular vision, who fail to identify the largest test disparity (e.g., 400 arcsecs in the clinical circle test or 3600 arcsecs in the Vivid Vision stereo test) may has a true stereo threshold that is higher than the measurement limit. These observations are therefore right-censored.

If we treated these censored values as the true measured value, the estimated regression slope would be biased. If we excluded the censored observations, we would severely reduce the sample size and introduce selection bias, underestimating the variance and overestimating the effect sizes. The Tobit Model (also known as a censored regression model, originally proposed by Tobin, 1958) provides the correct framework. It uses the information from the observed values (where the true threshold is known) and the censored values (where the true threshold is only known to be above the limit) to estimate the parameters of the underlying, latent, continuous relationship. Additionally, Stereo thresholds are typically non-normally distributed and highly skewed on the raw scale (arcseconds). To satisfy the normality assumptions underlying the Tobit model and stabilize variance, all threshold values were log-transformed prior to modeling.

*Model Specification for Assessing Stereo Improvement*

To evaluate whether Binocular Balance Improvement predicts Stereo Threshold Improvement, the model must account for each trainee’s initial ability. We therefore include the pre-training log-threshold as a covariate. The latent (true but partially unobserved) post-training log-threshold is defined as:

$Y_{post,log,i}^{latent}\text{=}\beta_{0}\text{+}\beta_{1}X_{i}\text{+}\beta_{2}Y_{pre,log,i}\text{+}\epsilon_{i},\text{ }\epsilon_{i}\sim N\left( 0,\sigma^{2} \right)$ (C1)

The observed post-training log-threshold ($Y_{post,log,i}$) is related to the latent log-threshold ($Y_{post,log,i}^{latent}$) by the right-censoring mechanism at the upper log-limit $U_{log}$:

$Y_{post,log,i}\text{=}\left\{ \begin{aligned} Y_{post,log,i}^{latent}\text{ if }Y_{post,log,i}^{latent}\text{<}U_{log}\text{ }\left( Observed \right) \\ U_{log}\text{ if }Y_{post,log,i}^{latent}\geq U_{log}\text{ }\left( Censored \right) \end{aligned} \right.$ (C2)

Where $X_{i}$ is the quantitative measure of Binocular Balance Improvement, given by Eq. B1, and $Y_{pre,log,i}$is the Pre-Training Log-Threshold (using the observed log-value, but $Y_{pre,log}\text{=}U_{log}$for unmeasurable pre-training thresholds).

*Model Fitting and Parameter Estimation*

The model parameters $\theta\text{=}\left[ \beta_{0},\beta_{1},\beta_{2},\sigma\right]$are estimated using Maximum Likelihood Estimation (MLE) (Greene, 2012). The log-likelihood function (L) is adapted for right-censoring at $U_{log}$. The function combines two components:

1. For Observed Data ($Y_{post,log,i}^{latent}\text{<}U_{log}$): The contribution is the log of the Probability Density Function (PDF) of the observation.

2. For Censored Data ($Y_{post,log,i}^{latent}\geq U_{log}$): The contribution is the log of the Cumulative Distribution Function (CDF) area, specifically

$log\left( P\left( Y_{post,log,i}^{latent}\geq U_{log} \right) \right)\text{=}log\left( 1\text{-}\Phi\left( \frac{\left( U_{log}\text{-}\mu_{i} \right)}{\sigma} \right) \right)$, (C3)

where $\Phi$ is the standard normal CDF and $\mu_{i}\text{=}\beta_{0}\text{+}\beta_{1}X_{i}\text{+}\beta_{2}Y_{pre,log,i}$.

The parameters are found by maximizing the total log-likelihood:

$\hat{\theta}\text{=}argmaxL\left( \theta\text{|}Y_{post,log},X,Y_{pre,log} \right)$ (C4)

In MATLAB, this was implemented using the fmincon optimization function to minimize the negative log-likelihood subject to the constraint that the standard deviation ($\sigma$) must be greater than zero.

*Interpretation of Model Parameters*

The estimated coefficients $\hat{\theta}\text{=}\left[ \hat{\beta_{0}},\hat{\beta_{1}},\hat{\beta_{2}},\hat{\sigma} \right]$ from the Tobit model describe effects on the latent, uncensored post-training log-threshold ($Y_{post,log}^{latent}$). Note that a **lower** threshold value indicates **better** stereo performance.

Intercept ($\hat{\beta_{0}}$) represents baseline latent post-training threshold when both predictors, Binocular Balance Improvement ($X$) and Pre-Training Log-Threshold ($Y_{pre,log}$), are zero. Slope for Binocular Balance Improvement ($\hat{\beta_{1}}$) is the key parameter of interest. A negative $\hat{\beta_{1}}$ indicates that greater improvement in binocular balance predicts better (lower) latent post-training stereo thresholds, independent of initial stereo ability. Slope for Pre-Training Threshold ($\hat{\beta_{2}}$) is expected to be positive: poorer initial stereoacuity predicts poorer post-training stereoacuity. Residual Standard Deviation ($\hat{\sigma}$) represents the remaining variability in latent stereo thresholds after accounting for both predictors.

Standard errors (SE) and p-values were computed from the inverse Hessian (the asymptotic covariance matrix), allowing Wald tests of the null hypothesis $H_{0}:\beta_{i}\text{=}0$.

*References*

Greene, W. H. (2012). Econometric Analysis (7th ed.). Boston, MA: Pearson Education

Tobin, J. (1958). *Estimation of relationships for limited dependent variables.* Econometrica: Journal of the Econometric Society, 24-36.
